# Relevance of national, regional and global virome projects on pandemics prediction, prevention, and control: a social network analysis of GVP-citing articles

**DOI:** 10.1590/0074-02760230116

**Published:** 2023-10-20

**Authors:** Bruna de Paula Fonseca, Carlos Medicis Morel

**Affiliations:** 1 Fundação Oswaldo Cruz-Fiocruz, Centro de Desenvolvimento Tecnológico em Saúde, Rio de Janeiro, RJ, Brasil; 2 Fundação Oswaldo Cruz-Fiocruz, Centro de Desenvolvimento Tecnológico em Saúde, Instituto Nacional de Ciência e Tecnologia de Inovação em Doenças de Populações Negligenciadas, Rio de Janeiro, RJ, Brasil

**Keywords:** global virome project, social network analysis, pandemics, public health

## Abstract

**BACKGROUND:**

The Global Virome Project (GVP) was proposed in 2018 as an evolution of the USAID PREDICT project and was presented as a “collaborative scientific initiative to discover zoonotic viral threats and stop future pandemics”. The immediate response was mixed, with public health and scientific communities representatives showing skepticism, if not direct opposition.

**OBJECTIVES:**

The economic, social, and health consequences of the coronavirus disease 2019 (COVID-19) pandemic demonstrated how unprepared the world was in the face of new pandemics. This paper analyses the impact of the GVP on the scientific and public health communities.

**METHODS:**

Published scientific articles that cited the two 2018 seminal publications proposing the project were analysed using social network analysis methods.

**FINDINGS:**

Encompassing the periods before and after the onset of the Covid-19 pandemic, the results indicate that (i) the concepts of the GVP have received more support than opposition in the scientific literature; (ii) its foundations should be updated to address the specific criticisms.

**MAIN CONCLUSIONS:**

Shifting focus to national virome projects can provide tangible, regional benefits that can positively contribute towards a consensus on achieving a high level of preparedness for the ever-present possibility of the following global viral pandemic.

The coronavirus disease 2019 (COVID-19) pandemic triggered the past century’s most significant global economic crisis. In 2020, the economic activity in 90 percent of countries contracted, the world economy shrank by about 3%, and global poverty increased for the first time in a generation.^1^ Predicting the next pandemic, “Disease X”, to prevent its sanitary and economic impact, represents a significant challenge to science and public health, demanding research to enhance pandemic preparedness, new technologies, and strategies.^2^ ^,^ ^3^ ^,^ ^4^

Prevention is not as great a priority in the health sector or any government health-associated agency when compared to direct medical services.^5^ Prediction, as a statement about the future, faces an even lower recognition in priorities as few scientific or technological studies dare to address the challenges of forecasting the probability of the next epidemic. A noteworthy exception is PREDICT, a project started in 2009 under the emerging pandemic threats (EPT) program of the United States Agency for International Development (USAID) led by the UC Davis One Health Institute.^6^

The PREDICT project led to the gathering of an invited group of specialists in 2016 at the Bellagio Conference Centre of the Rockefeller Foundation,^7^ which generated the scientific basis for the outline of the Global Virome Project (GVP). Proposed as an initiative to identify and characterise the vast array of viruses in the world, the project was structured to better understand virus diversity and their potential to impact humans, animals, and the environment. Using advanced technologies and collaborative efforts, the GVP is expected to collect and analyse samples from various ecosystems worldwide. This would allow for the identification of new viruses and risk assessments, two essential elements for the development of the tools needed to detect emerging viral threats and the pharmaceuticals to treat and prevent their associated diseases. The details of the GVP proposal were consolidated into two seminal papers published in 2018.^8^ ^,^ ^9^

GVP has been the subject of much controversy as some critics have raised concerns, given the sheer scale of the task at hand, about the feasibility and cost of the project.^10^ Despite these concerns, proponents of the GVP argue that it is a necessary step in safeguarding public health and preventing future outbreaks of deadly diseases. Similar initiatives in the past, such as the Human Genome Project, have yielded valuable insights into the workings of the human body and paved the way for new medical treatments. Ultimately, the debate over the GVP will likely continue for some time.

Here in this paper, we address the repercussions and impact of this proposed initiative on the scientific and public health communities using the tools of social network analysis (SNA). SNA has been widely used to address the citation patterns of an individual or a collection of papers. Citation networks are a crucial tool for understanding knowledge dynamics and can offer invaluable insights for quantitatively analysing the impact of specific scientific contributions.^11^ By examining the connections between scientific publications, citation networks allow us to trace ideas’ evolution and individual contributions’ influence. These networks offer a powerful means of evaluating the impact of scientific research. By analysing the number and quality of citations received by a particular paper, we can gauge its influence on the field and its potential for future impact.^11^

## MATERIALS AND METHODS

*Data* - Citation was used as a proxy for impact, and data was comprised of the number of scientific publications that cited one or both of the 2018 seminal papers proposing the GVP. Citation information was obtained from Scopus (Elsevier) and Web of Science (Clarivate) databases on December 1st, 2022 [see Supplementary data (Table I)]. The metadata associated with each publication, such as title, year, DOI, list of authors, journal, abstract, received citations, etc., were catalogued, and duplicates were removed based on DOI numbers. Full texts were obtained through institutional subscriptions or, when under paywalls, individual payments.

*Classification* - Citations usually correlate positively with impact, but negative citations are also an essential aspect of scholarly discourse as they contribute to the ongoing evaluation and refinement of scientific knowledge.^12^ Negative citations can challenge or refute the original work’s findings and highlight limitations or flaws in experimental design and conclusions. To most accurately address the impact of the GVP proposal, citing publications were categorised as Supporters, Opposers, or Neutrals in their position towards GVP and or its strategy according to the criteria specified in Table I [for examples, see Supplementary Data (Tables II-III)]. Two independent researchers analysed each citing publication for the sentences and context that meet the criteria. A third researcher reviewed discordant classifications and a final classification was agreed upon by consensus.

**TABLE I:** Classification criteria

Classification	Criteria
Opposer	Absence of consensus. Concluded no guarantees for a return on investments. Cited biosecurity risks. Discussed the technological challenges (infrastructure, equipment, personnel). Predicted high absolute costs. Suggested a low-cost benefit for human health. Highlighted evolutionary uncertainty.
Neutral	Reported results from Global Virome Project (GVP) initiatives. Single mentions.
Supporter	Advocated for the development of a reference database for rapid pathogen identifications and risk assessment. Corroborated GVP methodologies. Emphasised the importance of local, regional, or global surveillance efforts for prevention. Highlighted the importance of studying virus diversity and ecology as well as their potential and processes for emergence.

*Citation network* - A list of cited-and-citing relationships was imported into the Gephi software (v0.10.1) to build a directed egocentric network of the two GVP seminal papers. In the graphical representation of the networks, papers are nodes, and citations links between papers are directed edges between nodes (*i.e.*, the links have a direction, from a citing paper to the cited paper). The size of the nodes was proportional to the number of citations they received. Nodes were coloured in red (Opposer), green (Supporter), or blue (Neutral), according to their classification.

## RESULTS

**Fig. 1:**
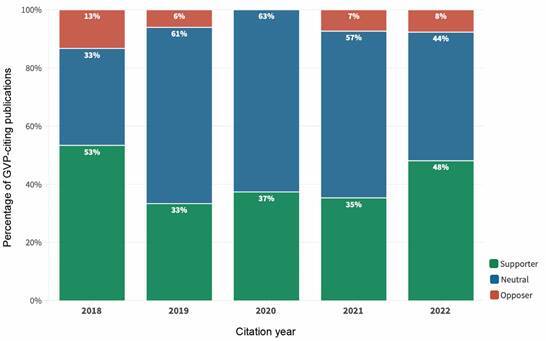
distribution of articles citing the two 2018 Global Virome Project (GVP) papers according to publication year and classification. For every year considered Opposers are the minority. Interesting to note that no Opposer article was published during the year the coronavirus disease 2019 (COVID-19) pandemic started.

The distribution of GVP-citing articles per year and classification are listed in Table II and shown in Fig. 1, based on our previous experience with the social network analysis methodology and approaches.^13^ ^,^ ^14^ ^,^ ^15^ ^,^ ^16^ Together, the two GVP seminal papers were cited by 243 papers. The majority of citations were made in 2020 (31%), followed by 2021 (28%) and 2022 (21%). In 2018, the year the GVP seminal papers were published, most citations had a supportive character, which declined in the following years and rose again in 2022. “Neutral” papers were in the majority from 2019 to 2021. Opposing papers maintained a relatively stable pattern, ranging from 13 to 7% throughout the years. It is interesting to note that no article classified as “Opposer” could be retrieved in 2020, the year the COVID-19 pandemic started.

**TABLE II:** Distribution of Global Virome Project (GVP) citing articles by year and classification. The detailed analysis of the 13 articles designated as GVP Opposers and examples from 24 of 96 articles classified as GVP Supporters are presented in Supplementary data (Tables II-III), respectively

	2018	2019	2020	2021	2022	Total
Opposers	2	2	0	5	4	13
Neutrals	5	20	47	39	23	134
Supporters	8	11	28	24	25	96
Total	15	33	75	68	52	243

**Fig. 2:**
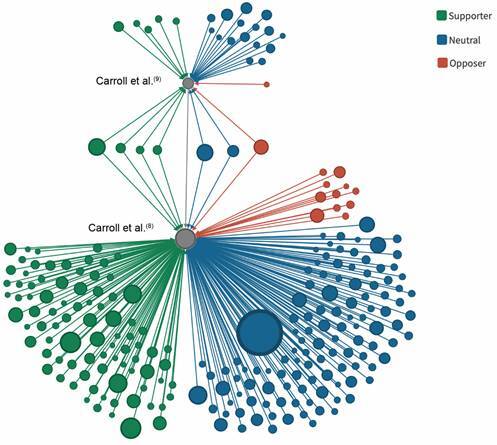
network of publications citing the Global Virome Project (GVP). Papers citing the two 2018 GVP^8^ ^,^ ^9^ articles were coloured according to our classification criteria as Supporters (green), Neutrals (blue), or Opposers (red). The diameter of nodes is proportional to the number of citations received by each article. The large circle in blue (neutral) is the paper by Gorbalenya et al.,(17) which received 3,951 citations. This paper was published by the Coronaviridae Study Group of the International Committee on Taxonomy of Viruses, classifying 2019-nCoV and naming it severe acute respiratory syndrome coronavirus 2 (SARS-CoV-2).

Fig. 2 represents an ego network analysis cantered on the two 2018 publications by Carroll et al. proposing the GVP.^8^ ^,^ ^9^ According to this figure, the Science paper had a larger impact as compared to the World Health Organization (WHO) Bulletin article: 214 publications cited the Science paper (88%), 22 cited the WHO Bulletin paper (9%), and seven cited both publications (3%). Opposer articles are minority compared to those supporting or having a neutral position concerning the GVP.

According to these results, the 2018 GVP proposal has received more support than opposition in the scientific literature five years after its launch. Many of the arguments of Opposers against the GVP, most raised before the COVID-19 pandemic hit the world, are now seen as not robust enough to undermine the GVP approach as a valid component of the toolbox to predict, prevent, and control pandemics.

## DISCUSSION

The global impact of COVID-19 turned the issues of pathogen discovery, infectious diseases surveillance, and pandemic prediction into a more acceptable and amenable subject to scientific and forecasting studies, as foreseen by some authors.^2^ ^,^ ^3^ ^,^ ^18^ In 2021, the National Science Foundation (NSF) launched a Call for Applications for Phase I of its Predictive Intelligence for Pandemic Prevention (PIPP) program, designed to “support research focused on addressing the prediction and prevention of infectious disease pandemics”.^19^ This demonstrates how outdated the 2018 motto “Pandemics: spend on surveillance, not prediction” became.^10^

In this Discussion, we address two main areas: firstly, we analyse and/or compare, from the citing articles, selected arguments pro- and against GVP. Secondly, we address where and how the GVP initial proposal would need to be updated, considering that we are now in a post-pandemic era, which is quite different from the world in 2018.

*Arguments against or in favour of GVP* - GVP high absolute costs: When launched in 2018, the GVP aimed at raising $1.2 billion US dollars within a 10-year time frame, and not from the Fiscal Year 2019 budget for the US National Institute of Allergy and Infectious Diseases (NIAID), as incorrectly published.^10^ This price tag, comparable to other ‘big science’ projects such as the Human Genome ($2.7 billion over 13 years), is dwarfed when compared to the economic losses inflicted by the COVID-19 pandemic in the world.

Firm support, in economic terms, of the GVP was raised by Fulci and collaborators:^20^ “Compared with the estimated 16 trillion USD economic losses of the current pandemic,^21^ a GVP would be worth being supported even if the best-expected outcome was shortening by 1 month the resolution of the next pandemic.”

*GVP and biosecurity risks* - The classification of severe acute respiratory syndrome coronavirus 2 (SARS-CoV-2) as a risk group 3 biological agent^22^ and the sporadic re-emergence of diseases caused by risk group 4 pathogens, such as Ebola, Lassa, Marburg, and Sabiá,^23^ ^,^ ^24^ has had as a consequence that “More countries are building high-containment laboratories, developing dual-use biotechnologies, and conducting risky research with pathogens”.^25^ As one of the authors pointed out (CMM), biosecurity facilities should be considered an integral part of any public health response to emerging infectious disease prevention, control, and management.^26^ Overplaying the potential dangers of biosecurity laboratories being built around the world for public health purposes could have unforeseen consequences: (i) strengthen a hidden agenda aimed at establishing authoritarian, top-down power to control these maximum security labs wherever they would be located;^25^ (ii) foster the forgetfulness that the greatest threat to biosecurity is the use of well-known biological agents in episodes of biological warfare, such as (a) the intentional spread of smallpox among Native American populations during the French and Indian War from 1754 and 1763 by a British commander;^27^ (b) the accidental escape of an aerosol of anthrax pathogen from a Soviet military facility leading to the Sverdlovsk anthrax outbreak of 1979;^28^ (c) the attack with threatening letters containing anthrax spores to US senators through the US Postal Service in 2001.^29^ A GVP coordination and management system could become essential in managing and improving the safety of BSL-3 and BSL-4 laboratories in public health networks.

*Development of a reference database* - A recurring argument among GVP Opposers relates to the high technological challenges ahead and the low-cost benefit for human health. The Supporters, on the contrary, advocate for the potential role of the GVP in developing a reference database for rapid pathogen identification and risk assessment. Both highlight the importance of understanding virus diversity and ecology.

Our ignorance of the animal virome is unanimously recognised by all virologists, Opposers, or Supporters, as we know less about its diversity than any other biological entity. Most documented animal viruses have been sampled from just two phyla - the Chordata and the Arthropoda.^30^ This underscores the importance of the discovery component of GVP since the birth of its ancestral program, the PREDICT project, considering the importance that viral discovery efforts could play in controlling future epidemics.^31^

*GVP today: an update?* - Although the GVP has been a work in progress since the origins of the PREDICT Project back in 2009, its formal launch can be assumed to be 2018, when Carroll and collaborators published their February and March articles.^8^ ^,^ ^9^ To properly evaluate the significance of the GVP, one has to consider that the world pre-and post the Covid-19 pandemic represent two quite different entities, as illustrated in documents from these two periods:

(i) September 2017: the Embassy of the United States of America in Beijing was among the strongest supporters of the GVP initiative, as described in the unclassified message available on the website of the organisation US Right To Know (USRTK), entitled “China’s Interest in the Global Virome Project Presents an Opportunity for Global Health Cooperation”;^32^

(ii) October 2022: the Minority Oversight Staff of the Senate Committee on Health Education, Labour, and Pensions publishes the Interim Report “An Analysis of the Origins of the COVID-19 Pandemic,” which concludes that “the COVID-19 pandemic was more likely than not, the result of a research-related incident” (at the Coronavirus Research in Wuhan and the Wuhan Institute of Virology).^33^

In this geopolitically tense world of today, the GVP, as proposed in its original format of 2018, would have no chance to globally mobilise countries, funding agencies, or the scientific and public health communities. To mend the damage due to the present split of previously cooperating parties, we propose two areas of work that could mobilise researchers and experts from interested countries to action:

*National virome projects* - A shift to a bottom-up approach, instead of top-down, could provide the “proof” that the objectives for the GVP are achievable and of discernible value. The initiation of national virome projects can have the power to re-mobilise countries, within the context of their citizens, to focus on predicting disease X, which could be a re-emerging pathogen such as the yellow fever virus in Brazil in 2016-2018.^34^ ^,^ ^35^ Each country would define its agenda and follow its regulatory norms. In the short term, this approach could stimulate the constitution of a scientific leadership that would strengthen networks of reference laboratories and positively impact national R&D capacity through investments in infrastructure and human resources. In the long-term, such programs could organically evolve to encompass regional partnerships, as pathogens do not respect nation boundaries, which could grow into a global policy.

*Discovery through mission-oriented research* - Virus discovery, characterisation, and taxonomy are critical areas of research that merit attention from the scientific and public health communities,^36^ as recognised even by authors who claim to be opposed to the GVP.^30^ ^,^ ^37^ The importance of basic research in virology needs to be correctly prioritised by funding agencies that prefer to support projects directed to short-term, more immediate goals. National virome projects would be able to recruit young scientists and talents calling for projects aiming at discovering and characterising viral populations in the rich fauna and flora of every country. Following national norms and guidelines, these projects could avoid the bureaucracy and politics of international science and technology funding agencies and international regulatory authorities.

*In conclusion* - Our social network analysis of papers discussing the GVP indicated that the GVP received more support than opposition from the scientific and health communities. While there are valid concerns to be addressed, the potential benefits of this ambitious undertaking are too great to ignore. Stakeholders must work together to ensure that any virome project adapts itself to the new reality of the present, the onset of COVID-19 has fractured the post-pandemic world we now live in. For this purpose, we propose that national and regional virome projects be considered as critical steps to establishing a cooperative approach to responsible and ethical research that can build towards a global consensus on how to deliver the ultimate goal of protecting the health and well-being of people around the world from disease X.
