# Supplementary material for: Relevance of national, regional and global virome projects on pandemics prediction, prevention, and control: a social network analysis of GVP-citing articles
Source: Mem Inst Oswaldo Cruz. 2023 Oct 20;118:e230116. doi: 10.1590/0074-02760230116 (PMC10599233; doi:10.1590/0074-02760230116)
Supplement: Supplementary file 1 [file 1678-8060-mioc-118-e230116-s.pdf]

TABLE I

Publications citing Global Virome Project (GVP) analysed

Authors	Title	Publication year	Journal	DOI
Goncalves, PCT; Moura, AS; Cordeiro, MNDS; Campos, P	Mr. Silva and Patient Zero: A Medical Social Network and Data Visualization Information System	2018	SIMULATION, IMAGE PROCESSING, AND ULTRASOUND SYSTEMS FOR ASSISTED DIAGNOSIS AND NAVIGATION	10.1007/978-3-030-01045-4_13
Perrings, C; Levin, S; Daszak, P	The Economics of Infectious Disease, Trade and Pandemic Risk	2018	ECOHEALTH	10.1007/s10393-018-1347-0
Wu, AP	Viral diseases meet omics: Time for systems virology	2018	SCIENCE CHINA-LIFE SCIENCES	10.1007/s11427-018-9397-2
Gao, GF	From A'' IV to Z''IKV: Attacks from Emerging and Re-emerging Pathogens	2018	CELL	10.1016/j.cell.2018.02.025
Wang, WQ; Han, GZ	The Expanding Diversity of RNA Viruses in Vertebrates	2018	TRENDS IN MICROBIOLOGY	10.1016/j.tim.2018.04.003
Schmidt, C	The virome hunters	2018	NATURE BIOTECHNOLOGY	10.1038/nbt.4268
Tetz, G; Tetz, V	Prion-like Domains in Eukaryotic Viruses	2018	SCIENTIFIC REPORTS	10.1038/s41598-018-27256-w
Woolhouse, M	Sources of human viruses	2018	SCIENCE	10.1126/science.aav4265
Wu, ZQ; Lu, L; Du, J; Yang, L; Ren, XW; Liu, B; Jiang, JY; Yang, J; Dong, J; Sun, LL; Zhu, YF; Li, YH; Zheng, DD; Zhang, C; Su, HX; Zheng, YT; Zhou, HN; Zhu, GJ; Li, HY; Chmura, A; Yang, F; Daszak, P; Wang, JW; Liu, QY; Jin, Q	Comparative analysis of rodent and small mammal viromes to better understand the wildlife origin of emerging infectious diseases	2018	MICROBIOME	10.1186/s40168-018-0554-9
Vasconcellos, AG; Fonseca, BDFE; Morel, CM	Revisiting the concept of Innovative Developing Countries (IDCs) for its relevance to health innovation and neglected tropical diseases and for the prevention and control of epidemics	2018	PLOS NEGLECTED TROPICAL DISEASES	10.1371/journal.pntd.0006469
Shillitoe E.J.	The Microbiome of Oral Cancer	2018	CRITICAL REVIEWS IN ONCOGENESIS	10.1615/CritRevOncog.2018027422
Carroll, D; Watson, B; Togami, E; Daszak, P; Mazet, JAK; Chrisman, CJ; Rubin, EM; Wolfe, N; Morel, CM; Gao, GF; Burci, GL; Fukuda, K; Auewarakul, P; Tomori, O	Building a global atlas of zoonotic viruses	2018	BULLETIN OF THE WORLD HEALTH ORGANIZATION	10.2471/BLT.17.205005
Pablos-Mendez, A; Raviglione, MC	A New World Health Era	2018	GLOBAL HEALTH-SCIENCE AND PRACTICE	10.9745/GHSP-D-17-00297
Yeager A.	Spotting Spillover	2018	SCIENTIST	N/A
Holmes, EC; Rambaut, A; Andersen, KG	Pandemics: spend on surveillance, not prediction	2018	NATURE	10.1038/d41586-018-05373-w
Atoni, E; Zhao, L; Karungu, S; Obanda, V; Agwanda, B; Xia, H; Yuan, ZM	The discovery and global distribution of novel mosquito-associated viruses in the last decade (2007-2017)	2019	REVIEWS IN MEDICAL VIROLOGY	10.1002/rmv.2079
Ibarra-Cerdeña C.N., González-Martínez A., Valdez-Tah A.R., Chi-Méndez C.G., Castillo-Burguete M.T., Ramsey J.M.	Tackling exposure to Chagas disease in the Yucatan from a human ecology perspective	2019	CULTURE, ENVIRONMENT AND HEALTH IN THE YUCATAN PENINSULA: A HUMAN ECOLOGY PERSPECTIVE	10.1007/978-3-030-27001-8_16
Sweeten, TL; Croen, LA; Windham, GC; Odell, JD; Stubbs, EG; Torres, AR	Brief Report: Low Rates of Herpesvirus Detection in Blood of Individuals with Autism Spectrum Disorder and Controls	2019	JOURNAL OF AUTISM AND DEVELOPMENTAL DISORDERS	10.1007/s10803-018-3691-x
Ellwanger J.H., Kaminski V.D.L., Chies J.A.B.	Emerging infectious disease prevention: Where should we invest our resources and efforts?	2019	JOURNAL OF INFECTION AND PUBLIC HEALTH	10.1016/j.jiph.2019.03.010
Xu J.	Reverse microbial etiology: A research field for predicting and preventing emerging infectious diseases caused by an unknown microorganism	2019	JOURNAL OF BIOSAFETY AND BIOSECURITY	10.1016/j.jobb.2018.12.005

Authors	Title	Publication year	Journal	DOI
Xue, L; Cai, WC; Gao, JS; Jiang, YT; Wu, HM; Zhang, L; Zuo, YT; Dong, RM; Pang, R; Zeng, HY; Wu, S; Wang, J; Zhang, JM; Wu, QP	Genome characteristics and molecular evolution of the human sapovirus variant GII.8	2019	INFECTION GENETICS AND EVOLUTION	10.1016/j.meegid.2019.05.017
Tan, ZZ; Yu, HJ; Xu, L; Zhao, ZH; Zhang, PS; Qu, YG; He, B; Tu, CC	Virome profiling of rodents in Xinjiang Uygur Autonomous Region, China: Isolation and characterization of a new strain of Wenzhou virus	2019	VIROLOGY	10.1016/j.virol.2019.01.010
Watanabe, T; Suzuki, N; Tomonaga, K; Sawa, H; Matsuura, Y; Kawaguchi, Y; Takahashi, H; Nagasaki, K; Kawaoka, Y	Neo-virology: The raison d'etre of viruses	2019	VIRUS RESEARCH	10.1016/j.virusres.2019.197751
Carlson, CJ; Zipfel, CM; Garnier, R; Bansal, S	Global estimates of mammalian viral diversity accounting for host sharing	2019	NATURE ECOLOGY & EVOLUTION	10.1038/s41559-019-0910-6
Fernandez-Correa, I; Truchado, DA; Gomez-Lucia, E; Domenech, A; Perez-Tris, J; Schmidt-Chanasit, J; Cadar, D; Benitez, L	A novel group of avian astroviruses from Neotropical passerine birds broaden the diversity and host range of Astroviridae	2019	SCIENTIFIC REPORTS	10.1038/s41598-019-45889-3
Jones, SD; Atshabar, B; Schmid, BV; Zuk, M; Amramina, A; Stenseth, NC	Living with plague: Lessons from the Soviet Union's antiplague system	2019	PROCEEDINGS OF THE NATIONAL ACADEMY OF SCIENCES OF THE UNITED STATES OF AMERICA	10.1073/pnas.1817339116
Lewnard, JA; Reingold, AL	Emerging Challenges and Opportunities in Infectious Disease Epidemiology	2019	AMERICAN JOURNAL OF EPIDEMIOLOGY	10.1093/aje/kwy264
Zhou, CLE; Malfatti, S; Kimbrel, J; Philipson, C; McNair, K; Hamilton, T; Edwards, R; Souza, B	multiPhATE: bioinformatics pipeline for functional annotation of phage isolates	2019	BIOINFORMATICS	10.1093/bioinformatics/btz258
Dallas, TA; Carlson, CJ; Poisot, T	Testing predictability of disease outbreaks with a simple model of pathogen biogeography	2019	ROYAL SOCIETY OPEN SCIENCE	10.1098/rsos.190883
Polonsky, JA; Baidjoe, A; Kamvar, ZN; Cori, A; Durski, K; Edmunds, WJ; Eggo, RM; Funk, S; Kaiser, L; Keating, P; de Waroux, OL; Marks, M; Moraga, P; Morgan, O; Nouvellet, P; Ratnayake, R; Roberts, CH; Whitworth, J; Jombart, T	Outbreak analytics: a developing data science for informing the response to emerging pathogens	2019	PHILOSOPHICAL TRANSACTIONS OF THE ROYAL SOCIETY B-BIOLOGICAL SCIENCES	10.1098/rstb.2018.0276
Becker, DJ; Washburne, AD; Faust, CL; Mordecai, EA; Plowright, RK	The problem of scale in the prediction and management of pathogen spillover	2019	PHILOSOPHICAL TRANSACTIONS OF THE ROYAL SOCIETY B-BIOLOGICAL SCIENCES	10.1098/rstb.2019.0224
Johnson, RI; Tachedjian, M; Clayton, BA; Layton, R; Bergfeld, J; Wang, LF; Marsh, GA	Characterization of Teviot virus, an Australian bat-borne paramyxovirus	2019	JOURNAL OF GENERAL VIROLOGY	10.1099/jgv.0.001214
Zhang, Z; Cai, ZN; Tan, ZY; Lu, CY; Jiang, TJ; Zhang, GH; Peng, YS	Rapid identification of human-infecting viruses	2019	TRANSBOUNDARY AND EMERGING DISEASES	10.1111/tbed.13314
Wheeler, DL; Scott, J; Dung, JKS; Johnson, DA	Evidence of a trans-kingdom plant disease complex between a fungus and plant-parasitic nematodes	2019	PLOS ONE	10.1371/journal.pone.0211508
Garcia-Lopez, R; Perez-Brocal, V; Moya, A	Beyond cells - The virome in the human holobiont	2019	MICROBIAL CELL	10.15698/mic2019.09.689
Titcomb, GC; Jerde, CL; Young, HS	High-Throughput Sequencing for Understanding the Ecology of Emerging Infectious Diseases at the Wildlife-Human Interface	2019	FRONTIERS IN ECOLOGY AND EVOLUTION	10.3389/fevo.2019.00126
Mubareka, S; Groulx, N; Savory, E; Cutts, T; Theriault, S; Scott, JA; Roy, CJ; Turgeon, N; Bryce, E; Astrakianakis, G; Kirychuk, S; Girard, M; Kobinger, G; Zhang, C; Duchaine, C	Bioaerosols and Transmission, a Diverse and Growing Community of Practice	2019	FRONTIERS IN PUBLIC HEALTH	10.3389/fpubh.2019.00023

Authors	Title	Publication year	Journal	DOI
Connor, R; Brister, R; Buchmann, JP; Deboutte, W; Edwards, R; Marti-Carreras, J; Tisza, M; Zalunin, V; Andrade-Martinez, J; Cantu, A; D'Amour, M; Efremov, A; Fleischmann, L; Forero-Junco, L; Garmaeva, S; Giluso, M; Glickman, C; Henderson, M; Kellman, B; Kristensen, D; Leubsdorf, C; Levi, K; Levi, S; Pakala, S; Peddu, V; Ponsero, A; Ribeiro, E; Roy, F; Rutter, L; Saha, S; Shakya, M; Shean, R; Miller, M; Tully, B; Turkington, C; Youens-Clark, K; Vanmechelen, B; Busby, B	NCBI's Virus Discovery Hackathon: Engaging Research Communities to Identify Cloud Infrastructure Requirements	2019	GENES	10.3390/genes10090714
Duarte, MA; Silva, JMF; Brito, CR; Teixeira, DS; Melo, FL; Ribeiro, BM; Nagata, T; Campos, FS	Faecal Virome Analysis of Wild Animals from Brazil	2019	VIRUSES-BASEL	10.3390/v11090803
Prada, D; Boyd, V; Baker, ML; O'Dea, M; Jackson, B	Viral Diversity of Microbats within the South West Botanical Province of Western Australia	2019	VIRUSES-BASEL	10.3390/v11121157
Asalone, KC; Nelson, MM; Bracht, JR	Novel Sequence Discovery by Subtractive Genomics	2019	JOVE-JOURNAL OF VISUALIZED EXPERIMENTS	10.3791/58877
Bhatia, R	Implementation framework for One Health approach	2019	INDIAN JOURNAL OF MEDICAL RESEARCH	10.4103/ijmr.IJMR_1517_18
Manuguerra J.-C.	Les chainons de l'émergence des maladies virales chez l'homme, du passage zoonotique à la transmission interhumaine efficace : les exemples du syndrome respiratoire aigu sévère (SARS) et de la maladie à virus Ebola	2019	BULLETIN DE L'ACADEMIE VETERINAIRE DE FRANCE	10.4267/2042/70854
Gao G.F.	For a better world: Biosafety strategies to protect global health	2019	BIOSAFETY AND HEALTH	10.1016/j.bsheal.2019.03.001
Jonas, O; Seifman, R	Do we need a Global Virome Project?	2019	LANCET GLOBAL HEALTH	10.1016/S2214-109X(19)30335-3
Warren, CJ; Sawyer, SL	How host genetics dictates successful viral zoonosis	2019	PLOS BIOLOGY	10.1371/journal.pbio.3000217
Dehghan, S; Seto, J; Liu, EB; Ismail, AM; Madupu, R; Heim, A; Jones, MS; Dyer, DW; Chodosh, J; Seto, D	A Zoonotic Adenoviral Human Pathogen Emerged through Genomic Recombination among Human and Nonhuman Simian Hosts	2019	JOURNAL OF VIROLOGY	10.1128/JVI.00564-19
Sommer, J; Trautner, C; Witte, AK; Fister, S; Schoder, D; Rossmanith, P; Mester, PJ	Don't Shut the Stable Door after the Phage Has BoltedThe Importance of Bacteriophage Inactivation in Food Environments	2019	VIRUSES-BASEL	10.3390/v11050468
Watanabe, T; Kawaoka, Y	Villains or heroes? The raison d'etre of viruses	2020	CLINICAL & TRANSLATIONAL IMMUNOLOGY	10.1002/cti2.1114
Heaton, SM	Frontiers in antiviral therapy and immunotherapy	2020	CLINICAL & TRANSLATIONAL IMMUNOLOGY	10.1002/cti2.1115
Cobas D., Mäkinen V., Rossi M.	Tailoring r-index for Document Listing Towards Metagenomics Applications	2020	LECTURE NOTES IN COMPUTER SCIENCE (INCLUDING SUBSERIES LECTURE NOTES IN ARTIFICIAL INTELLIGENCE AND LECTURE NOTES IN BIOINFORMATICS)	10.1007/978-3-030-59212-7_21
Skern, T	Editorial: Archives of Virology's 80th anniversary symposium-6 months later	2020	ARCHIVES OF VIROLOGY	10.1007/s00705-020-04678-4
Evans, TS; Shi, ZL; Boots, M; Liu, WJ; Olival, KJ; Xiao, XM; Vandewoude, S; Brown, H; Chen, JL; Civitello, DJ; Escobar, L; Grohn, Y; Li, HY; Lips, K; Liu, QY; Lu, JH; Martinez-Lopez, B; Shi, JS; Shi, XL; Xu, B; Yuan, LH; Zhu, GQ; Getz, WM	Synergistic China-US Ecological Research is Essential for Global Emerging Infectious Disease Preparedness	2020	ECOHEALTH	10.1007/s10393-020-01471-2

Authors	Title	Publication year	Journal	DOI
Yeh Y.-T., Swaminathan V., Terrones M.	The application of low-dimensional materials in virology and in the study of living organisms	2020	SYNTHESIS, MODELLING AND CHARACTERIZATION OF 2D MATERIALS AND THEIR HETEROSTRUCTURES	10.1016/B978-0-12-818475-2.00018-0
Mattei, JF	The links between epidemics and the environment	2020	BULLETIN DE L ACADEMIE NATIONALE DE MEDECINE	10.1016/j.banm.2020.06.008
Carlson, CJ; Hopkins, S; Bell, KC; Dona, J; Godfrey, SS; Kwak, ML; Lafferty, KD; Moir, ML; Speer, KA; Strona, G; Torchin, M; Wood, CL	A global parasite conservation plan	2020	BIOLOGICAL CONSERVATION	10.1016/j.biocon.2020.108596
Daszak P., Olival K.J., Li H.	A strategy to prevent future epidemics similar to the 2019-nCoV outbreak	2020	BIOSAFETY AND HEALTH	10.1016/j.bsheal.2020.01.003
Sempowski, GD; Saunders, KO; Acharya, P; Wiehe, KJ; Haynes, BF	Pandemic Preparedness: Developing Vaccines and Therapeutic Antibodies For COVID-19	2020	CELL	10.1016/j.cell.2020.05.041
Morens, DM; Fauci, AS	Emerging Pandemic Diseases: How We Got to COVID-19	2020	CELL	10.1016/j.cell.2020.08.021
Stensmyr, MC	Mosquito Biology: How a Quest for Water Spawned a Thirst for Blood	2020	CURRENT BIOLOGY	10.1016/j.cub.2020.07.030
Kenyon, C	Emergence of zoonoses such as COVID-19 reveals the need for health sciences to embrace an explicit eco-social conceptual framework of health and disease	2020	EPIDEMICS	10.1016/j.epidem.2020.100410
Suman, R; Javaid, M; Haleem, A; Vaishya, R; Bahl, S; Nandan, D	Sustainability of Coronavirus on Different Surfaces	2020	JOURNAL OF CLINICAL AND EXPERIMENTAL HEPATOLOGY	10.1016/j.jceh.2020.04.020
Peters, A; Vetter, P; Guitart, C; Lotfinejad, N; Pittet, D	Understanding the emerging coronavirus: what it means for health security and infection prevention	2020	JOURNAL OF HOSPITAL INFECTION	10.1016/j.jhin.2020.02.023
Zhang, JH; Ma, KX; Li, HA; Liao, M; Qi, WB	The continuous evolution and dissemination of 2019 novel human coronavirus	2020	JOURNAL OF INFECTION	10.1016/j.jinf.2020.02.001
Sun, MQ; Wang, H; Li, XY	Modification of cellulose microfibers by polyglutamic acid and mesoporous silica nanoparticles for Enterovirus 71 adsorption	2020	MATERIALS LETTERS	10.1016/j.matlet.2020.128320
Zhu, PF; Garber, PA; Wang, L; Li, M; Belov, K; Gillespie, TR; Zhou, XM	Comprehensive Knowledge of Reservoir Hosts is Key to Mitigating Future Pandemics	2020	INNOVATION	10.1016/j.xinn.2020.100065
Kenyon, C	Syndemic responses to COVID-19 should include an ecological dimension	2020	LANCET	10.1016/S0140-6736(20)32219-4
Albery, GF; Eskew, EA; Ross, N; Olival, KJ	Predicting the global mammalian viral sharing network using phylogeography	2020	NATURE COMMUNICATIONS	10.1038/s41467-020-16153-4
Latinne, A; Hu, B; Olival, KJ; Zhu, GJ; Zhang, LB; Li, HY; Chmura, AA; Field, HE; Zambrana-Torrelio, C; Epstein, JH; Li, B; Zhang, W; Wang, LF; Shi, ZL; Daszak, P	Origin and cross-species transmission of bat coronaviruses in China	2020	NATURE COMMUNICATIONS	10.1038/s41467-020-17687-3
Gorbalenya, AE; Baker, SC; Baric, RS; de Groot, RJ; Drosten, C; Gulyaeva, AA; Haagmans, BL; Lauber, C; Leontovich, AM; Neuman, BW; Penzar, D; Perlman, S; Poon, LLM; Samborskiy, DV; Sidorov, IA; Sola, I; Ziebuhr, J	The species Severe acute respiratory syndrome-related coronavirus: classifying 2019-nCoV and naming it SARS-CoV-2	2020	NATURE MICROBIOLOGY	10.1038/s41564-020-0695-z
Martinez, JM; Martinez-Hernandez, F; Martinez-Garcia, M	Single-virus genomics and beyond	2020	NATURE REVIEWS MICROBIOLOGY	10.1038/s41579-020-00444-0
Letko, M; Seifert, SN; Olival, KJ; Plowright, RK; Munster, VJ	Bat-borne virus diversity, spillover and emergence	2020	NATURE REVIEWS MICROBIOLOGY	10.1038/s41579-020-0394-z

Authors	Title	Publication year	Journal	DOI
Bellantuono, L; Monaco, A; Tangaro, S; Amoroso, N; Aquaro, V; Bellotti, R	An equity-oriented rethink of global rankings with complex networks mapping development	2020	SCIENTIFIC REPORTS	10.1038/s41598-020-74964-3
Yeh, YT; Gulino, K; Zhanga, YH; Sabestien, A; Chou, TW; Zhou, B; Lin, Z; Albert, I; Lu, HG; Swaminathan, V; Ghedin, E; Terrones, M	A rapid and label-free platform for virus capture and identification from clinical samples	2020	PROCEEDINGS OF THE NATIONAL ACADEMY OF SCIENCES OF THE UNITED STATES OF AMERICA	10.1073/pnas.1910113117
Kress, WJ; Mazet, JAK; Hebert, PDN	Intercepting pandemics through genomics	2020	PROCEEDINGS OF THE NATIONAL ACADEMY OF SCIENCES OF THE UNITED STATES OF AMERICA	10.1073/pnas.2009508117
Heymann, J; Raub, A; Waisath, W; McCormack, M; Weistroffer, R; Moreno, G; Wong, EL; Earle, A	Protecting health during COVID-19 and beyond: A global examination of paid sick leave design in 193 countries*	2020	GLOBAL PUBLIC HEALTH	10.1080/17441692.2020.1764076
Davies, A; Hooks, G; Knox-Hayes, J; Lievanos, RS	Riskscapes and the socio-spatial challenges of climate change	2020	CAMBRIDGE JOURNAL OF REGIONS ECONOMY AND SOCIETY	10.1093/cjres/rsaa016
Malukiewicz J., Boere V., De Oliveira M.A.B., D'arc M., Ferreira J.V.A., French J., Housman G., De Souza C.I., Jerusalinsky L., De Melo F.R., Valenca-Montenegro M.M., Moreira S.B., Silva I.D.O.E., Pacheco F.S., Rogers J., Pissinatti A., Del Rosario R.C.H., Ross C., Ruiz-Miranda C.R., Pereira L.C.M., Schiel N., Da Silva F.D.F.R., Souto A., Šlipogor V., Tardif S.	An Introduction to the Callithrix Genus and Overview of Recent Advances in Marmoset Research	2020	ILAR JOURNAL	10.1093/ilar/ilab027
Zorzal, PB; Hauegen, RC; Pimenta, FP	Biodiversity and the patent system: the Brazilian case	2020	JOURNAL OF INTELLECTUAL PROPERTY LAW & PRACTICE	10.1093/jiplp/jpaa125
Wei, XM; Li, X; Cui, J	Evolutionary perspectives on novel coronaviruses identified in pneumonia cases in China	2020	NATIONAL SCIENCE REVIEW	10.1093/nsr/nwaa009
Paskey, AC; Ng, JHJ; Rice, GK; Chia, WN; Philipson, CW; Foo, RJH; Cer, RZ; Long, KA; Lueder, MR; Frey, KG; Hamilton, T; Mendenhall, IH; Smith, GJ; Wang, LF; Bishop-Lilly, KA	The temporal RNA virome patterns of a lesser dawn bat (Eonycteris spelaea) colony revealed by deep sequencing	2020	VIRUS EVOLUTION	10.1093/ve/veaa017
Qian, XB; Chen, T; Xu, YP; Chen, L; Sun, FX; Lu, MP; Liu, YX	A guide to human microbiome research: study design, sample collection, and bioinformatics analysis	2020	CHINESE MEDICAL JOURNAL	10.1097/CM9.0000000000000871
Rajagopal, K; Keller, SP; Akkanti, B; Bime, C; Loyalka, P; Cheema, FH; Zwischenberger, JB; El Banayosy, A; Pappalardo, F; Slaughter, MS; Slepian, MJ	Advanced Pulmonary and Cardiac Support of COVID-19 Patients: Emerging Recommendations From ASAIO-A Living Working Document	2020	ASAIO JOURNAL	10.1097/MAT.0000000000001180
Carlson, CJ; Dallas, TA; Alexander, LW; Phelan, AL; Phillips, AJ	What would it take to describe the global diversity of parasites?	2020	PROCEEDINGS OF THE ROYAL SOCIETY B-BIOLOGICAL SCIENCES	10.1098/rspb.2020.1841
Wells, K; Morand, S; Wardeh, M; Baylis, M	Distinct spread of DNA and RNA viruses among mammals amid prominent role of domestic species	2020	GLOBAL ECOLOGY AND BIOGEOGRAPHY	10.1111/geb.13045
Colunga-Salas, P; Sanchez-Montes, S; Grostieta, E; Verde-Arregoitia, LD; Cabrera-Garrido, MY; Becker, I; Leon-Paniagua, L	What do studies in wild mammals tell us about human emerging viral diseases in Mexico?	2020	TRANSBOUNDARY AND EMERGING DISEASES	10.1111/tbed.13336
Dobson, AP; Pimm, SL; Hannah, L; Kaufman, L; Ahumada, JA; Ando, AW; Bernstein, A; Busch, J; Daszak, P; Engelmann, J; Kinnaird, MF; Li, BBV; Loch-Temzelides, T; Lovejoy, T; Nowak, K; Roehrdanz, PR; Vale, MM	Ecology and economics for pandemic prevention Investments to prevent tropical deforestation and to limit wildlife trade will protect against future zoonosis outbreaks	2020	SCIENCE	10.1126/science.abc3189

Authors	Title	Publication year	Journal	DOI
Xue, QH; Xu, HK; Liu, HD; Pan, JJ; Yang, J; Sun, M; Chen, YF; Xu, WW; Cai, XP; Ma, HW	Epitope-Containing Short Peptides Capture Distinct IgG Serodynamics That Enable Differentiating Infected from Vaccinated Animals for Live-Attenuated Vaccines	2020	JOURNAL OF VIROLOGY	10.1128/JVI.01573-19
Morens, DM; Daszak, P; Markel, H; Taubenberger, JK	Pandemic COVID-19 Joins History's Pandemic Legion	2020	MBIO	10.1128/mBio.00812-20
Li, B; Si, HR; Zhu, Y; Yang, XL; Anderson, DE; Shi, ZL; Wang, LF; Zhou, P	Discovery of Bat Coronaviruses through Surveillance and Probe Capture-Based Next-Generation Sequencing	2020	MSPHERE	10.1128/mSphere.00807-19
Clarkson, MC; Aguas, R; Sweet, K; Roberts, T; Strobel, M; Newton, PN	How many human pathogens are there in Laos? An estimate of national human pathogen diversity and analysis of historical trends	2020	BMJ GLOBAL HEALTH	10.1136/bmjgh-2020-002972
Sanz, CL; Pessoa, M	Images of the future. Risk and responsibilization in neoliberal management of the future	2020	TEMPO SOCIAL	10.11606/0103-2070.ts.2020.160462
Rajagopal, K; Keller, SP; Akkanti, B; Bime, C; Loyalka, P; Cheema, FH; Zwischenberger, JB; El Banayosy, A; Pappalardo, F; Slaughter, MS; Slepian, MJ	Advanced Pulmonary and Cardiac Support of COVID-19 Patients Emerging Recommendations From ASAIO-a Living Working Document	2020	CIRCULATION-HEART FAILURE	10.1161/IRCHEARTFAILURE.120.007175
Nakatani, H; Katsuno, K; Urabe, H	Global health landscape challenges triggered by COVID-19	2020	INFLAMMATION AND REGENERATION	10.1186/s41232-020-00144-5
Brett, TS; Rohani, P	Dynamical footprints enable detection of disease emergence	2020	PLOS BIOLOGY	10.1371/journal.pbio.3000697
Olival, KJ; Cryan, PM; Amman, BR; Baric, RS; Blehert, DS; Brook, CE; Calisher, CH; Castle, KT; Coleman, JTH; Daszak, P; Epstein, JH; Field, H; Frick, WF; Gilbert, AT; Hayman, DTS; Ip, HS; Karesh, WB; Johnson, CK; Kading, RC; Kingston, T; Lorch, JM; Mendenhall, IH; Peel, AJ; Phelps, KL; Plowright, RK; Reeder, DM; Reichard, JD; Sleeman, JM; Streicker, DG; Towner, JS; Wang, LF	Possibility for reverse zoonotic transmission of SARS-CoV-2 to free-ranging wildlife: A case study of bats	2020	PLOS PATHOGENS	10.1371/journal.ppat.1008758
Nambiar P.	India to Envision One Health Movement for Confronting Emerging Health Threats: From Concept to Approach Toward Institutionalization	2020	INTERNATIONAL JOURNAL OF ONE HEALTH	10.14202/IJOH.2020.165-176
Lucas J.A.	Foresight Versus Hindsight? That is the Question	2020	OUTLOOKS ON PEST MANAGEMENT	10.1564/v31_aug_01
Grande-Lopez, V	The teachings of the epidemic thrillers to the study of the COVID-19	2020	REVISTA ESPANOLA DE COMUNICACION EN SALUD	10.20318/recs.2020.5442
Wannous, C	Climate change and other risk drivers of animal health and zoonotic disease emergencies: the need for a multidisciplinary and multisectoral approach to disaster risk management	2020	REVUE SCIENTIFIQUE ET TECHNIQUE-OFFICE INTERNATIONAL DES EPIZOOTIES	10.20506/rst.39.2.3097
Mian, M; Talada, J; Klobas, A; Torres, S; Rasheed, Y; Javed, H; Lughmani, Z; Forough, R	A customized program for the identification of conserved protein sequence motifs	2020	BIOTECHNIQUES	10.2144/btn-2019-0039
Howard, G; Bartram, J; Brocklehurst, C; Colford, JM; Costa, F; Cunliffe, D; Dreibelbis, R; Eisenberg, JNS; Evans, B; Girones, R; Hrudey, S; Willetts, J; Wright, CY	COVID-19: urgent actions, critical reflections and future relevance of 'WaSH': lessons for the current and future pandemics	2020	JOURNAL OF WATER SANITATION AND HYGIENE FOR DEVELOPMENT / JOURNAL OF WATER AND HEALTH	10.2166/washdev.2020.218 / 10.2166/wh.2020.162
Minakshi, P; Ghosh, M; Kumar, R; Brar, B; Lambe, UP; Banerjee, S; Ranjan, K; Kumar, B; Goel, P; Malik, YS; Prasad, G	An Insight to Nanamenicinal Approaches to Combat Viral Zoonoses	2020	CURRENT TOPICS IN MEDICINAL CHEMISTRY	10.2174/1568026620666200325114400
Taha, AE	The Severe Acute Respiratory Syndrome Coronavirus-2 Pandemic: An Overview to Control Human-wildlife and Human-human Interactions	2020	JOURNAL OF PURE AND APPLIED MICROBIOLOGY	10.22207/JPAM.14.2.02

Authors	Title	Publication year	Journal	DOI
Balykova L.A., Granovskaya M.V., Zaslavskaya K.Y., Simakina E.N., Agaf'ina A.S., Ivanova A.Y., Kolontarev K.B., Pushkar D.Y.		2020	INFECTIOUS DISEASES: NEWS, OPINIONS, TRAINING	10.33029/2305-3496-2020-9-3-16-29
Walzer, C	COVID-19 and the Curse of Piecemeal Perspectives	2020	FRONTIERS IN VETERINARY SCIENCE	10.3389/fvets.2020.582983
Truchado, DA; Llanos-Garrido, A; Oropesa-Olmedo, DA; Cerrada, B; Cea, P; Moens, MAJ; Gomez-Lucia, E; Domenech, A; Mila, B; Perez-Tris, J; Cadar, D; Benitez, L	Comparative Metagenomics of Palearctic and Neotropical Avian Cloacal Viromes Reveal Geographic Bias in Virus Discovery	2020	MICROORGANISMS	10.3390/microorganisms8121869
Yeh, KB; Fair, JM; Smith, W; Torres, TM; Lucas, J; Monagin, C; Winegar, R; Fletcher, J	Assessing Climate Change Impact on Ecosystems and Infectious Disease: Important Roles for Genomic Sequencing and a One Health Perspective	2020	TROPICAL MEDICINE AND INFECTIOUS DISEASE	10.3390/tropicalmed5020090
Kwok, KTT; Nieuwenhuijse, DF; Phan, MVT; Koopmans, MPG	Virus Metagenomics in Farm Animals: A Systematic Review	2020	VIRUSES-BASEL	10.3390/v12010107
Songchun Y., Jun L., Liming L.	[New progress in epidemiological research]	2020	CHINESE JOURNAL OF ENDEMIOLOGY	10.3760/cma.j.issn.0254-6450.2020.01.001
Bhatia, R; Abraham, P	Time to revisit national response to pandemics	2020	INDIAN JOURNAL OF MEDICAL RESEARCH	10.4103/ijmr.IJMR_846_20
Morens, DM; Breman, JG; Calisher, CH; Doherty, PC; Hahn, BH; Keusch, GT; Kramer, LD; LeDuc, JW; Monath, TP; Taubenberger, JK	The Origin of COVID-19 and Why It Matters	2020	AMERICAN JOURNAL OF TROPICAL MEDICINE AND HYGIENE	10.4269/ajtmh.20-0849
Pierce, L; Snyder, JA	Historical Origins of Firm Ownership Structure: The Persistent Effects of the African Slave Trade	2020	ACADEMY OF MANAGEMENT JOURNAL	10.5465/amj.2018.0597
Mina, MJ; Metcalf, CJE; McDermott, AB; Douek, DC; Farrar, J; Grenfell, BT	Science Forum: A Global lmmunological Observatory to meet a time of pandemics	2020	ELIFE	10.7554/eLife.58989
Hie B., Zhong E.D., Bryson B.D., Berger B.	Learning Mutational Semantics	2020	ADVANCES IN NEURAL INFORMATION PROCESSING SYSTEMS	N/A
Grover, G; Magan, R	Estimation of Quality Adjusted Life Year (QALY) for Different States of India During COVID-19	2020	STATISTICS AND APPLICATIONS	N/A
Mollentze, N; Streicker, DG	Viral zoonotic risk is homogenous among taxonomic orders of mammalian and avian reservoir hosts	2020	PROCEEDINGS OF THE NATIONAL ACADEMY OF SCIENCES OF THE UNITED STATES OF AMERICA	10.1073/pnas.1919176117
Chatterjee, P; Nagi, N; Agarwal, A; Das, B; Banerjee, S; Sarkar, S; Gupta, N; Gangakhedkar, RR	The 2019 novel coronavirus disease (COVID-19) pandemic: A review of the current evidence	2020	INDIAN JOURNAL OF MEDICAL RESEARCH	10.4103/ijmr.IJMR_519_20
Tambo, E; El-Dessouky, AG; Khater, EIM; Xianonng, Z	Enhanced surveillance and response approaches for pilgrims and local Saudi populations against emerging Nipah, Zika and Ebola viral diseases outbreaks threats	2020	JOURNAL OF INFECTION AND PUBLIC HEALTH	10.1016/j.jiph.2020.01.313
Gomez, GF; Isaza, JP; Segura, JA; Alzate, JF; Gutierrez, LA	Metatranscriptomic virome assessment of Rhipicephalus microplus from Colombia	2020	TICKS AND TICK-BORNE DISEASES	10.1016/j.ttbdis.2020.101426
Asiri, YI; Alsayari, A; Muhsinah, AB; Mabkhot, YN; Hassan, MZ	Benzothiazoles as potential antiviral agents	2020	JOURNAL OF PHARMACY AND PHARMACOLOGY	10.1111/jphp.13331
Minakshi, P; Ghosh, M; Kumar, R; Brar, B; Lambe, UP; Banerjee, S; Ranjan, K; Kumar, B; Goel, P; Malik, YS; Prasad, G	An Insight to Nanamenicinal Approaches to Combat Viral Zoonoses	2020	CURRENT TOPICS IN MEDICINAL CHEMISTRY	10.2174/1568026620666200325114400

Authors	Title	Publication year	Journal	DOI
Bhatia, R	Need for integrated surveillance at human-animal interface for rapid detection response to emerging coronavirus infections using One Health approach	2020	INDIAN JOURNAL OF MEDICAL RESEARCH	10.4103/ijmr.IJMR_623_20
Tyshkovskiy, A; Panchin, AY	There is no evidence of SARS-CoV-2 laboratory origin: Response to Segreto and Deigin	2021	BIOESSAYS	10.1002/bies.202000325
Tyshkovskiy, A; Panchin, AY	There is still no evidence of SARS-CoV-2 laboratory origin: Response to Segreto and Deigin	2021	BIOESSAYS	10.1002/bies.202100194
Ghale-Noie, ZN; Salmaninejad, A; Bergquist, R; Mollazadeh, S; Hoseini, B; Sahebkar, A	Genetic Aspects and Immune Responses in Covid-19: Important Organ Involvement.	2021	IDENTIFCATION OF BIOMARKERS, NEW TREATMENTS, AND VACCINES FOR COVID-19	10.1007/978-3-030-71697-4_1
Lv, ZM; Xiong, DW; Shi, JC; Long, M; Chen, ZL	The Interaction Between Viruses and Intestinal Microbiota: A Review	2021	CURRENT MICROBIOLOGY	10.1007/s00284-021-02623-5
Lu, CY; Peng, YS	Computational Viromics: Applications of the Computational Biology in Viromics Studies	2021	VIROLOGICA SINICA	10.1007/s12250-021-00395-7
Pyle J.D., Whelan S.P.J., Bloyet L.-M.	Structure and function of negative-strand RNA virus polymerase complexes	2021	ENZYMES	10.1016/bs.enz.2021.09.002
Souza T.M.L., Morel C.M.	The COVID-19 pandemics and the relevance of biosafety facilities for metagenomics surveillance, structured disease prevention and control	2021	BIOSAFETY AND HEALTH	10.1016/j.bsheal.2020.11.007
Mohan S.V., Hemalatha M., Kopperi H., Ranjith I., Kumar A.K.	SARS-CoV-2 in environmental perspective: Occurrence, persistence, surveillance, inactivation and challenges	2021	CHEMICAL ENGINEERING JOURNAL	10.1016/j.cej.2020.126893
Nishikiori, M; Ahlquist, P	Transmembrane redox regulation of genome replication functions in positive-strand RNA viruses	2021	CURRENT OPINION IN VIROLOGY	10.1016/j.coviro.2020.12.003
Shivaprakash, KN; Sen, S; Paul, S; Kiesecker, JM; Bawa, KS	Mammals, wildlife trade, and the next global pandemic	2021	CURRENT BIOLOGY	10.1016/j.cub.2021.06.006
Vicenti, I; Martina, MG; Boccuto, A; De Angelis, M; Giavarini, G; Dragoni, F; Marchi, S; Trombetta, CM; Crespan, E; Maga, G; Eydoux, C; Decroly, E; Montomoli, E; Nencioni, L; Zazzi, M; Radi, M	System-oriented optimization of multi-target 2,6-diaminopurine derivatives: Easily accessible broad-spectrum antivirals active against flaviviruses, influenza virus and SARS-CoV-2	2021	EUROPEAN JOURNAL OF MEDICINAL CHEMISTRY	10.1016/j.ejmech.2021.113683
Glennon, EE; Bruijning, M; Lessler, J; Miller, IF; Rice, BL; Thompson, RN; Wells, K; Metcalf, CJE	Challenges in modeling the emergence of novel pathogens	2021	EPIDEMICS	10.1016/j.epidem.2021.100516
Hilderink, MH; de Winter, II	No need to beat around the bushmeat-The role of wildlife trade and conservation initiatives in the emergence of zoonotic diseases	2021	HELIYON	10.1016/j.heliyon.2021.e07692
Kawang, K; Naoudom, U; Kowitdamrong, E; Kerr, SJ; Ruxrungtham, K; Nilaratanakul, V	Sensitivity and specificity of anti-double-stranded RNA immunofluorescence for universal detection of viral infection in respiratory specimens	2021	HELIYON	10.1016/j.heliyon.2021.e08471
Buchy P., Buisson Y., Cintra O., Dwyer D.E., Nissen M., Ortiz de Lejarazu R., Petersen E.	COVID-19 pandemic: lessons learned from more than a century of pandemics and current vaccine development for pandemic control	2021	INTERNATIONAL JOURNAL OF INFECTIOUS DISEASES	10.1016/j.ijid.2021.09.045
Rice, BL; Douek, DC; McDermott, AB; Grenfell, BT; Metcalf, CJE	Why are there so few (or so many) circulating coronaviruses?	2021	TRENDS IN IMMUNOLOGY	10.1016/j.it.2021.07.001
Li Z., Jiang J., Ruan X., Tong Y., Xu S., Han L., Xu J.	The zoonotic and natural foci characteristics of SARS-CoV-2	2021	JOURNAL OF BIOSAFETY AND BIOSECURITY	10.1016/j.jobb.2021.06.002

Authors	Title	Publication year	Journal	DOI
Frutos, R; Gavotte, L; Devaux, CA	Understanding the origin of COVID-19 requires to change the paradigm on zoonotic emergence from the spillover to the circulation model	2021	INFECTION GENETICS AND EVOLUTION	10.1016/j.meegid.2021.104812
Otte, J; Pica-Ciamarra, U	Emerging infectious zoonotic diseases: The neglected role of food animals	2021	ONE HEALTH	10.1016/j.onehlt.2021.100323
Albery, GF; Becker, DJ	Fast-lived Hosts and Zoonotic Risk	2021	TRENDS IN PARASITOLOGY	10.1016/j.pt.2020.10.012
Plowright, RK; Reaser, JK; Locke, H; Woodley, SJ; Patz, JA; Becker, DJ; Oppler, G; Hudson, PJ; Tabor, GM	Land use-induced spillover: a call to action to safeguard environmental, animal, and human health	2021	LANCET PLANETARY HEALTH	10.1016/S2542-5196(21)00031-0
Forbes, KM; Anzala, O; Carlson, CJ; Kelvin, AA; Kuppalli, K; Leroy, EM; Maganga, GD; Masika, MM; Mombo, IM; Mwaengo, DM; Niama, RF; Nziza, J; Ogola, J; Pickering, BS; Rasmussen, AL; Sironen, T; Vapalahti, O; Webala, PW; Kindrachuk, J	Towards a coordinated strategy for intercepting human disease emergence in Africa	2021	LANCET MICROBE	10.1016/S2666-5247(20)30220-2
Albery, GF; Becker, DJ; Brierley, L; Brook, CE; Christofferson, RC; Cohen, LE; Dallas, TA; Eskew, EA; Fagre, A; Farrell, MJ; Glennon, E; Guth, S; Joseph, MB; Mollentze, N; Neely, BA; Poisot, T; Rasmussen, AL; Ryan, SJ; Seifert, S; Sjodin, AR; Sorrell, EM; Carlson, CJ	The science of the host-virus network	2021	NATURE MICROBIOLOGY	10.1038/s41564-021-00999-5
Fisher, MC; Murray, KA	Emerging infections and the integrative environment-health sciences: the road ahead	2021	NATURE REVIEWS MICROBIOLOGY	10.1038/s41579-021-00510-1
Oppenheim, B; Brown, K; Waldman, R	The world needs an intergovernmental panel on pandemic risk	2021	NATURE MEDICINE	10.1038/s41591-021-01374-x
Guo, Q; Li, M; Wang, CH; Guo, JY; Jiang, XQ; Tan, J; Wu, SF; Wang, PH; Xiao, TT; Zhou, M; Fang, ZC; Xiao, YH; Zhu, HQ	Predicting hosts based on early SARS-CoV-2 samples and analyzing the 2020 pandemic	2021	SCIENTIFIC REPORTS	10.1038/s41598-021-96903-6
Slagman, S; Fessner, WD	Biocatalytic routes to anti-viral agents and their synthetic intermediates	2021	CHEMICAL SOCIETY REVIEWS	10.1039/d0cs00763c
Gerberding, JL; Haynes, BF	Vaccine Innovations - Past and Future	2021	NEW ENGLAND JOURNAL OF MEDICINE	10.1056/NEJMp2029466
Grange, ZL; Goldstein, T; Johnson, CK; Anthony, S; Gilardi, K; Daszak, P; Olival, KJ; O'Rourke, T; Murray, S; Olson, SH; Togami, E; Vidal, G; Panel, E; Mazet, JAK	Ranking the risk of animal-to-human spillover for newly discovered viruses	2021	PROCEEDINGS OF THE NATIONAL ACADEMY OF SCIENCES OF THE UNITED STATES OF AMERICA	10.1073/pnas.2002324118
Van Oosterhout, C	Mitigating the threat of emerging infectious diseases; a coevolutionary perspective	2021	VIRULENCE	10.1080/21505594.2021.1920741
Fay E.J., Balla K.M., Roach S.N., Shepherd F.K., Putri D.S., Wiggen T.D., Goldstein S.A., Pierson M.J., Ferris M.T., Thefaine C.E., Tucker A., Salnikov M., Cortez V., Compton S.R., Kotenko S.V., Hunter R.C., Masopust D., Elde N.C., Langlois R.A.	Natural rodent model of viral transmission reveals biological features of virus population dynamics	2021	JOURNAL OF EXPERIMENTAL MEDICINE	10.1084/jem.20211220
Herrmann C., Cadwell K.	Playing dirty with virus transmission	2021	JOURNAL OF EXPERIMENTAL MEDICINE	10.1084/jem.20212358
Fulci, V; Carissimi, C; Laudadio, I	COVID-19 and Preparing for Future Ecological Crises: Hopes from Metagenomics in Facing Current and Future Viral Pandemic Challenges	2021	OMICS-A JOURNAL OF INTEGRATIVE BIOLOGY	10.1089/omi.2021.0058
Zhou, CLE; Kimbrel, J; Edwards, R; McNair, K; Souza, BA; Malfatti, S	MultiPhATE2: code for functional annotation and comparison of phage genomes	2021	G3-GENES GENOMES GENETICS	10.1093/g3journal/jkab074

Authors	Title	Publication year	Journal	DOI
Dye C.	The great health dilemma: is prevention better than cure?	2021	OXFORD UNIVERSITY PRESS	10.1093/ oso/9780198853824.001.0001
Schreiber, SJ; Ke, RA; Loverdo, C; Park, M; Ahsan, P; Lloyd-Smith, JO	Cross-scale dynamics and the evolutionary emergence of infectious diseases	2021	VIRUS EVOLUTION	10.1093/ve/veaa105
Santos, PD; Ziegler, U; Szillat, KP; Szentiks, CA; Strobel, B; Skuballa, J; Merbach, S; Grothmann, P; Tews, BA; Beer, M; Hoper, D	In action-an early warning system for the detection of unexpected or novel pathogens	2021	VIRUS EVOLUTION	10.1093/ve/veab085
Huang, S; Farrell, M; Stephens, PR	Infectious disease macroecology: parasite diversity and dynamics across the globe	2021	PHILOSOPHICAL TRANSACTIONS OF THE ROYAL SOCIETY B-BIOLOGICAL SCIENCES	10.1098/rstb.2020.0350
Carlson, CJ; Farrell, MJ; Grange, Z; Han, BA; Mollentze, N; Phelan, AL; Rasmussen, AL; Albery, GF; Bett, B; Brett-Major, DM; Cohen, LE; Dallas, T; Eskew, EA; Fagre, AC; Forbes, KM; Gibb, R; Halabi, S; Hammer, CC; Katz, R; Kindrachuk, J; Muylaert, RL; Nutter, FB; Ogola, J; Olival, KJ; Rourke, M; Ryan, SJ; Ross, N; Seifert, SN; Sironen, T; Standley, CJ; Taylor, K; Venter, M; Webala, PW	The future of zoonotic risk prediction	2021	PHILOSOPHICAL TRANSACTIONS OF THE ROYAL SOCIETY B-BIOLOGICAL SCIENCES	10.1098/rstb.2020.0358
Petrovan, SO; Aldridge, DC; Bartlett, H; Bladon, AJ; Booth, H; Broad, S; Broom, DM; Burgess, ND; Cleaveland, S; Cunningham, AA; Ferri, M; Hinsley, A; Hua, FY; Hughes, AC; Jones, K; Kelly, M; Mayes, G; Radakovic, M; Ugwu, CA; Uddin, N; Verissimo, D; Walzer, C; White, TB; Wood, JL; Sutherland, WJ	Post COVID-19: a solution scan of options for preventing future zoonotic epidemics	2021	BIOLOGICAL REVIEWS	10.1111/brv.12774
Govender, KN	Precision Pandemic Preparedness: Improving Diagnostics with Metagenomics	2021	JOURNAL OF CLINICAL MICROBIOLOGY	10.1128/JCM.02146-20
Kawasaki, J; Kojima, S; Tomonaga, K; Horie, M	Hidden Viral Sequences in Public Sequencing Data and Warning for Future Emerging Diseases	2021	MBIO	10.1128/mBio.01638-21
Musunuri, S; Sandbrink, JB; Monrad, JT; Palmer, MJ; Koblentz, GD	Rapid Proliferation of Pandemic Research: Implications for Dual-Use Risks	2021	MBIO	10.1128/mBio.01864-21
Carroll, D; Morzaria, S; Briand, S; Johnson, CK; Morens, D; Sumption, K; Tomori, O; Wacharphaueasadee, S	Preventing the next pandemic: the power of a global viral surveillance network	2021	BMJ-BRITISH MEDICAL JOURNAL	10.1136/bmj.n485
Deng, Z; Delwart, E	ContigExtender: a new approach to improving de novo sequence assembly for viral metagenomics data	2021	BMC BIOINFORMATICS	10.1186/s12859-021-04038-2
Wu, ZQ; Han, YL; Liu, B; Li, HY; Zhu, GJ; Latinne, A; Dong, J; Sun, LL; Su, HX; Liu, LG; Du, J; Zhou, SY; Chen, MX; Kritiyakan, A; Jittapalapong, S; Chaisiri, K; Buchy, P; Duong, V; Yang, JA; Jiang, JY; Xu, X; Zhou, HN; Yang, F; Irwin, DM; Morand, S; Daszak, P; Wang, JW; Jin, Q	Decoding the RNA viromes in rodent lungs provides new insight into the origin and evolutionary patterns of rodent-borne pathogens in Mainland Southeast Asia	2021	MICROBIOME	10.1186/s40168-020-00965-z
Mollentze, N; Babayan, SA; Streicker, DG	Identifying and prioritizing potential human-infecting viruses from their genome sequences	2021	PLOS BIOLOGY	10.1371/journal.pbio.3001390
Brierley, L; Fowler, A	Predicting the animal hosts of coronaviruses from compositional biases of spike protein and whole genome sequences through machine learning	2021	PLOS PATHOGENS	10.1371/journal.ppat.1009149
Tilak R., Bhattacharya S., Tilak V.W., Sinha S.	Disease X: Exploring the Unexplored, Knowable Unknown	2021	JOURNAL OF COMMUNICABLE DISEASES	10.24321/0019.5138.202183

Authors	Title	Publication year	Journal	DOI
Machalaba, C; Uhart, M; Ryser-Degiorgis, MP; Karesh, WB	Gaps in health security related to wildlife and environment affecting pandemic prevention and preparedness, 2007-2020	2021	BULLETIN OF THE WORLD HEALTH ORGANIZATION	10.2471/BLT.20.272690
Xiao, WM; Ma, ZS	Inter-Individual Diversity Scaling Analysis of the Human Virome With Classic Diversity-Area Relationship (DAR) Modeling	2021	FRONTIERS IN GENETICS	10.3389/fgene.2021.627128
Piret, J; Boivin, G	Pandemics Throughout History	2021	FRONTIERS IN MICROBIOLOGY	10.3389/fmicb.2020.631736
Keatts, LO; Robards, M; Olson, SH; Hueffer, K; Insley, SJ; Joly, DO; Kutz, S; Lee, DS; Chetkiewicz, CLB; Lair, S; Preston, ND; Pruvot, M; Ray, JC; Reid, D; Sleeman, JM; Stimmelmayr, R; Stephen, C; Walzer, C	Implications of Zoonoses From Hunting and Use of Wildlife in North American Arctic and Boreal Biomes: Pandemic Potential, Monitoring, and Mitigation	2021	FRONTIERS IN PUBLIC HEALTH	10.3389/fpubh.2021.627654
Liu, SS; Li, QY; Chu, XT; Zeng, MX; Liu, MB; He, XM; Zou, H; Zheng, JH; Corpe, C; Zhang, XY; Xu, JQ; Wang, J	Monitoring Coronavirus Disease 2019: A Review of Available Diagnostic Tools	2021	FRONTIERS IN PUBLIC HEALTH	10.3389/fpubh.2021.672215
Apakupakul, K; Deem, SL; Maqsood, R; Sithiyopasakul, P; Wang, DV; Lim, ES	Endogenization of a Prosimian Retrovirus during Lemur Evolution	2021	VIRUSES-BASEL	10.3390/v13030383
Tirera, S; de Thoisy, B; Donato, D; Bouchier, C; Lacoste, V; Franc, A; Lavergne, A	The Influence of Habitat on Viral Diversity in Neotropical Rodent Hosts	2021	VIRUSES-BASEL	10.3390/v13091690
Bhatia, R	Addressing challenge of zoonotic diseases through One Health approach	2021	INDIAN JOURNAL OF MEDICAL RESEARCH	10.4103/ijmr.IJMR_374_21
Chatterjee, P; Nair, P; Chersich, M; Terefe, Y; Chauhan, AS; Quesada, F; Simpson, G	One Health, Disease X & the challenge of Unknown Unknowns	2021	INDIAN JOURNAL OF MEDICAL RESEARCH	10.4103/ijmr.IJMR_601_21
Daszak, P	Lessons from COVID-19 to Help Prevent Future Pandemics	2021	CHINA CDC WEEKLY	10.46234/ccdcw2021.035
Seal, S; Dharmarajan, G; Khan, I	Evolution of pathogen tolerance and emerging infections: A missing experimental paradigm	2021	ELIFE	10.7554/eLife.68874
Ghislain, S	Animal Welfare in Trade Policy After the Covid-19 Pandemic: Another Set of learnings	2021	GLOBAL TRADE AND CUSTOMS JOURNAL	10.54648/gtcj2021033
Liu, L; Shen, Q; Li, N; He, YW; Han, N; Wang, XY; Meng, JX; Peng, YS; Pan, M; Jin, YT; Jiang, TJ; Tan, WJ; Wang, JL; Wu, AP	Comparative viromes of Culicoides and mosquitoes reveal their consistency and diversity in viral profiles	2021	BRIEFINGS IN BIOINFORMATICS	10.1093/bib/bbaa323
Pyle J.D., Whelan S.P.J., Bloyet L.-M.	Structure and function of negative-strand RNA virus polymerase complexes	2021	ENZYMES	10.1016/bs.enz.2021.09.002
Gilbert, W; Thomas, LF; Coyne, L; Rushton, J	Review: Mitigating the risks posed by intensification in livestock production: the examples of antimicrobial resistance and zoonoses	2021	ANIMAL	10.1016/j.animal.2020.100123
Liu, RR; Ma, RX; Liu, ZY; Hu, HF; Shu, JY; Hu, PZ; Kang, JJ; Zhang, YS; Han, MW; Zhang, XX; Zheng, YT; Ying, QK; Hou, SY; Wang, WQ; Wang, F; Cheng, N; Zhuang, Y; Lian, JQ; Jin, X; Wu, XA	HTNV infection of CD8(+) T cells is associated with disease progression in HFRS patients	2021	COMMUNICATIONS BIOLOGY	10.1038/s42003-021-02182-2
Warwick C., Steedman C.	Wildlife-pet markets in a one-health context	2021	INTERNATIONAL JOURNAL OF ONE HEALTH	10.14202/IJOH.2021.42-64
Ergunay, K	New viruses on the rise: a One Health and ecosystem-based perspective on emerging viruses	2021	FUTURE VIROLOGY	10.2217/fvl-2021-0215

Authors	Title	Publication year	Journal	DOI
Prakash, S; Srivastava, R; Coulon, PG; Dhanushkodi, NR; Chentoufi, AA; Tifrea, DF; Edwards, RA; Figueroa, CJ; Schubl, SD; Hsieh, L; Buchmeier, MJ; Bouziane, M; Nesburn, AB; Kuppermann, BD; BenMohamed, L	Genome-Wide B Cell, CD4(+), and CD8(+) T Cell Epitopes That Are Highly Conserved between Human and Animal Coronaviruses, Identified from SARS-CoV-2 as Targets for Preemptive Pan-Coronavirus Vaccines	2021	JOURNAL OF IMMUNOLOGY	10.4049/jimmunol.2001438
Werner, CS; Kasan, K; Geyer, JK; Elmasri, M; Farrell, MJ; Nunn, CL	Using phylogeographic link-prediction in primates to prioritize human parasite screening	2022	AMERICAN JOURNAL OF BIOLOGICAL ANTHROPOLOGY	10.1002/ajpa.24604
Shahzad, S; Willcox, M	The Possible Role of Prion-Like Viral Protein Domains on the Emergence of Novel Viruses as SARS-CoV-2	2022	JOURNAL OF MOLECULAR EVOLUTION	10.1007/s00239-022-10054-4
Berry, K; Horan, RD; Finnoff, D; Pompa, R; Daszak, P	Investing to Both Prevent and Prepare for COVID-XX	2022	ECOHEALTH	10.1007/s10393-022-01576-w
Daszak, P	International Collaboration is the Only Way to Protect Ourselves from the Next Pandemic	2022	ECOHEALTH	10.1007/s10393-022-01609-4
Almulhim T.S., Barahona I.	Decision support system for ranking relevant indicators for reopening strategies following COVID-19 lockdowns	2022	QUALITY AND QUANTITY	10.1007/s11135-021-01129-3
Destoumieux-Garzon, D; Matthies-Wiesler, F; Bierne, N; Binot, A; Boissier, J; Devouge, A; Garric, J; Gruetzmacher, K; Grunau, C; Guegan, JF; Hurtrez-Bousses, S; Huss, A; Morand, S; Palmer, C; Sarigiannis, D; Vermeulen, R; Barouki, R	Getting out of crises: Environmental, social-ecological and evolutionary research is needed to avoid future risks of pandemics	2022	ENVIRONMENT INTERNATIONAL	10.1016/j.envint.2021.106915
Frutos, R; Pliez, O; Gavotte, L; Devaux, CA	There is no origin to SARS-CoV-2	2022	ENVIRONMENTAL RESEARCH	10.1016/j.envres.2021.112173
Borghi-Silva, A; Back, GD; de Araujo, ASG; Oliveira, MR; Goulart, CD; Silva, RN; Bassi, D; Mendes, RG; Arena, R	COVID-19 seen from a syndemic perspective: Impact of unhealthy habits and future perspectives to combat these negative interactions in Latin America	2022	PROGRESS IN CARDIOVASCULAR DISEASES	10.1016/j.pcad.2022.04.006
Zhou, XX; Li, ZP; Zhang, Z; Zhu, LB; Liu, QJ	A rapid and label-free platform for virus enrichment based on electrostatic microfluidics	2022	TALANTA	10.1016/j.talanta.2021.122989
Forni D., Cagliani R., Clerici M., Sironi M.	Disease-causing human viruses: novelty and legacy	2022	TRENDS IN MICROBIOLOGY	10.1016/j.tim.2022.07.002
Bovo, S; Schiavo, G; Bolner, M; Ballan, M; Fontanesi, L	Mining livestock genome datasets for an unconventional characterization of animal DNA viromes	2022	GENOMICS	10.1016/j.ygeno.2022.110312
Jing, WY; Han, HS	Droplet Microfluidics for High-Resolution Virology	2022	ANALYTICAL CHEMISTRY	10.1021/acs.analchem.2c00615
Makin, S	Could an algorithm predict the next pandemic?	2022	NATURE	10.1038/d41586-022-03358-4
Guo, J; Huang, XL; Zhang, CX; Huang, PD; Li, YH; Wen, F; Wang, XJ; Yang, NS; Xu, M; Bi, YF; Ning, G; Li, L; Wang, WQ; Cao, YN	The blood virome of 10,585 individuals from the ChinaMAP	2022	CELL DISCOVERY	10.1038/s41421-022-00476-1
Sanchez, CA; Li, HY; Phelps, KL; Zambrana-Torrelio, C; Wang, LF; Zhou, P; Shi, ZL; Olival, KJ; Daszak, P	A strategy to assess spillover risk of bat SARS-related coronaviruses in Southeast Asia	2022	NATURE COMMUNICATIONS	10.1038/s41467-022-31860-w
Ko, KKK; Chng, KR; Nagarajan, N	Metagenomics-enabled microbial surveillance	2022	NATURE MICROBIOLOGY	10.1038/s41564-022-01089-w
Edgar, RC; Taylor, J; Lin, V; Altman, T; Barbera, P; Meleshko, D; Lohr, D; Novakovsky, G; Buchfink, B; Al-Shayeb, B; Banfield, JF; de la Pena, M; Korobeynikov, A; Chikhi, R; Babaian, A	Petabase-scale sequence alignment catalyses viral discovery	2022	NATURE	10.1038/s41586-021-04332-2
Carlson C.J., Albery G.F., Merow C., Trisos C.H., Zipfel C.M., Eskew E.A., Olival K.J., Ross N., Bansal S.	Climate change increases cross-species viral transmission risk	2022	NATURE	10.1038/s41586-022-04788-w
Zhou, XX; Zhu, LB; Li, WH; Liu, QJ	An integrated microfluidic chip for alginate microsphere generation and 3D cell culture	2022	ANALYTICAL METHODS	10.1039/d1ay01820e

Authors	Title	Publication year	Journal	DOI
Lewin, HA; Richards, S; Aiden, EL; Allende, ML; Archibald, JM; Balint, M; Barker, KB; Baumgartner, B; Belov, K; Bertorelle, G; Blaxter, ML; Cai, J; Caperello, ND; Carlson, K; Castilla-Rubio, JC; Chaw, SM; Chen, L; Childers, AK; Coddington, JA; Conde, DA; Corominas, M; Crandall, KA; Crawford, AJ; DiPalma, F; Durbin, R; Ebenezer, TE; Edwards, SV; Fedrigo, O; Flicek, P; Formenti, G; Gibbs, RA; Gilbert, MTP; Goldstein, MM; Graves, JM; Greely, HT; Grigoriev, IV; Hackett, KJ; Hall, N; Haussler, D; Helgen, KM; Hogg, CJ; Isobe, S; Jakobsen, KS; Janke, A; Jarvis, ED; Johnson, WE; Jones, SJM; Karlsson, EK; Kersey, PJ; Kim, JH; Kress, WJ; Kuraku, S; Lawniczak, MKN; Leebens-Mack, JH; Li, XY; Lindblad-Toh, K; Liu, X; Lopez, JV; Marques-Bonet, T; Mazard, S; Mazet, JAK; Mazzoni, CJ; Myers, EW; O'Neill, RJ; Paez, S; Park, H; Robinson, GE; Roquet, C; Ryder, OA; Sabir, JSM; Shaffer, HB; Shank, TM; Sherkow, JS; Soltis, PS; Tang, BP; Tedersoo, L; Uliano-Silva, M; Wang, K; Wei, XF; Wetzer, R; Wilson, JL; Xu, X; Yang, HM; Yoder, AD; Zhang, GJ	The Earth BioGenome Project 2020: Starting the clock	2022	PROCEEDINGS OF THE NATIONAL ACADEMY OF SCIENCES OF THE UNITED STATES OF AMERICA	10.1073/pnas.2115635118
Keusch G.T., Amuasi J.H., Anderson D.E., Daszak P., Eckerle I., Field H., Koopmans M., Lam S.K., Das Neves C.G., Peiris M., Perlman S., Wacharapluesadee S., Yadana S., Saif L.	Pandemic origins and a One Health approach to preparedness and prevention: Solutions based on SARS-CoV-2 and other RNA viruses	2022	PROCEEDINGS OF THE NATIONAL ACADEMY OF SCIENCES OF THE UNITED STATES OF AMERICA	10.1073/pnas.2202871119
Ye, SF; Lu, CY; Qiu, Y; Zheng, HP; Ge, XY; Wu, AP; Xia, ZX; Jiang, TJ; Zhu, HZ; Peng, YS	An atlas of human viruses provides new insights into diversity and tissue tropism of human viruses	2022	BIOINFORMATICS	10.1093/bioinformatics/btac275
Case, NT; Berman, J; Blehert, DS; Cramer, RA; Cuomo, C; Currie, CR; Ene, IV; Fisher, MC; Fritz-Laylin, LK; Gerstein, AC; Glass, NL; Gow, NAR; Gurr, SJ; Hittinger, CT; Hohl, TM; Iliev, ID; James, TY; Jin, HL; Klein, BS; Kronstad, JW; Lorch, JM; McGovern, V; Mitchell, AP; Segre, JA; Shapiro, RS; Sheppard, DC; Sil, A; Stajich, JE; Stukenbrock, EE; Taylor, JW; Thompson, D; Wright, GD; Heitman, J; Cowen, LE	The future of fungi: threats and opportunities	2022	G3-GENES GENOMES GENETICS	10.1093/g3journal/jkac224
Weems, EI; De la Sancha, NU; Anderson, LJ; Zambrana-Torrelio, C; Ferraris, RP	Centering Microbes in the Emerging Role of Integrative Biology in Understanding Environmental Change	2022	INTEGRATIVE AND COMPARATIVE BIOLOGY	10.1093/icb/icab047
Gibb, R; Albery, GF; Mollentze, N; Eskew, EA; Brierley, L; Ryan, SJ; Seifert, SN; Carlson, CJ	Mammal virus diversity estimates are unstable due to accelerating discovery effort	2022	BIOLOGY LETTERS	10.1098/rsbl.2021.0427
Hafezi, R; Asemi, P	Global scenarios under crises: the case of post COVID-19 era	2022	FORESIGHT	10.1108/FS-12-2021-0248
Kawasaki, J; Tomonaga, K; Horie, M	Large-scale investigation of zoonotic viruses in the era of high-throughput sequencing	2022	MICROBIOLOGY AND IMMUNOLOGY	10.1111/1348-0421.13033
Heyvaert, V	Governing Intersystemic Systemic Risks: Lessons from Covid and Climate Change	2022	MODERN LAW REVIEW	10.1111/1468-2230.12720
Bernstein, AS; Ando, AW; Loch-Temzelides, T; Vale, MM; Li, BV; Li, HY; Busch, J; Chapman, CA; Kinnaird, M; Nowak, K; Castro, MC; Zambrana-Torrelio, C; Ahumada, JA; Xiao, LY; Roehrdanz, P; Kaufman, L; Hannah, L; Daszak, P; Pimm, SL; Dobson, AP	The costs and benefits of primary prevention of zoonotic pandemics	2022	SCIENCE ADVANCES	10.1126/sciadv.abl4183

Authors	Title	Publication year	Journal	DOI
Sun, Y; Qu, Y; Yan, X; Yan, G; Chen, J; Wang, G; Zhao, Z; Liu, Y; Tu, C; He, B	Comprehensive Evaluation of RNA and DNA Viromic Methods Based on Species Richness and Abundance Analyses Using Marmot Rectal Samples	2022	MSYSTEMS	10.1128/msystems.00430-22
Kenyon, C; Vanbaelen, T; Van Dijck, C	Recent insights suggest the need for the STI field to embrace a more eco-social conceptual framework: A viewpoint	2022	INTERNATIONAL JOURNAL OF STD & AIDS	10.1177/09564624211064133
Kuchinski, KS; Duan, J; Himsworth, C; Hsiao, WL; Prystajecky, NA	ProbeTools: designing hybridization probes for targeted genomic sequencing of diverse and hypervariable viral taxa	2022	BMC GENOMICS	10.1186/s12864-022-08790-4
Woolaston, K; Nay, Z; Baker, ML; Brockett, C; Bruce, M; Degeling, C; Gilbert, J; Jackson, B; Johnson, H; Peel, A; Sahibzada, S; Oskam, C; Hewitt, CL	An argument for pandemic risk management using a multidisciplinary One Health approach to governance: an Australian case study	2022	GLOBALIZATION AND HEALTH	10.1186/s12992-022-00850-4
Lanfear J., Waterhouse R.M., Adam-Blondon A.-F., Agosti D., Baldrian P., Balech B., Corre E., Davey R.P., Lantz H., Pesole G., Quast C., Glöckner F.O., Raes N., Sandionigi A., Santamaria M., Addink W., Vohradsky J., Nunes-Jorge A., Willassen N.P.	Recommendations for connecting molecular sequence and biodiversity research infrastructures through ELIXIR	2022	F1000RESEARCH	10.12688/f1000research.73825.2
Bakacs T., Sandig V., Slavin S., Gumrukcu S., Hardy D., Renz W., Kovesdi I.	A clinically validated, broadly active, oral viral superinfection therapy could mitigate symptoms in early-stage COVID-19 patients	2022	INFECTIOUS DISORDERS - DRUG TARGETS	10.2174/18715265226662204191 30403
Herrero-Cofreces, S; Mougeot, F; Sironen, T; Meyer, H; Rodriguez-Pastor, R; Luque-Larena, JJ	Viral Zoonoses in Small Wild Mammals and Detection of Hantavirus, Spain	2022	EMERGING INFECTIOUS DISEASES	10.3201/eid2806.212508
Chong L.C., Khan A.M.	Historical milestone in 42 years of viral sequencing—Impetus for a community-driven sequencing of global priority pathogens	2022	FRONTIERS IN MICROBIOLOGY	10.3389/fmicb.2022.1020148
Zhu, WT; Yang, J; Lu, S; Jin, D; Pu, J; Wu, SS; Luo, XL; Liu, LY; Li, ZJ; Xu, JG	RNA Virus Diversity in Birds and Small Mammals From Qinghai-Tibet Plateau of China	2022	FRONTIERS IN MICROBIOLOGY	10.3389/fmicb.2022.780651
Sharshov, K	Avian Pathogens: Editorial and the Perspectives of Research	2022	MICROORGANISMS	10.3390/microorganisms10030543
Ponte, JMS; Seca, AML; Barreto, MC	Asparagopsis Genus: What We Really Know About Its Biological Activities and Chemical Composition	2022	MOLECULES	10.3390/molecules27061787
Edridge, AWD; Abd-Elfarag, G; Deijs, M; Jebbink, MF; van Hensbroek, MB; van der Hoek, L	Divergent Rhabdovirus Discovered in a Patient with New-Onset Nodding Syndrome	2022	VIRUSES-BASEL	10.3390/v14020210
Quer, J; Colomer-Castell, S; Campos, C; Andres, C; Pinana, M; Cortese, MF; Gonzalez-Sanchez, A; Garcia-Cehic, D; Ibanez, M; Pumarola, T; Rodriguez-Frias, F; Anton, A; Tabernero, D	Next-Generation Sequencing for Confronting Virus Pandemics	2022	VIRUSES-BASEL	10.3390/v14030600
Zhang F., Chase-Topping M., Guo C.-G., Woolhouse M.E.J.	Predictors of human-infective RNA virus discovery in the United States, China, and Africa, an ecological study	2022	ELIFE	10.7554/eLife.72123
Davies, K; Lim, M; Qin, TB; Riordan, P	CHANS-Law: preventing the next pandemic through the integration of social and environmental law	2022	INTERNATIONAL ENVIRONMENTAL AGREEMENTS-POLITICS LAW AND ECONOMICS	10.1007/s10784-022-09566-7
Sanyal, A; Agarwal, S; Ramakrishnan, U; Garg, KM; Chattopadhyay, B	Using Environmental Sampling to Enable Zoonotic Pandemic Preparedness	2022	JOURNAL OF THE INDIAN INSTITUTE OF SCIENCE	10.1007/s41745-022-00322-z
Wilk-da-Silva, R; Mederiros-Sousa, AR; Loporta, GZ; Mucci, LF; Prist, PR; Marrelli, MT	The influence of landscape structure on the dispersal pattern of yellow fever virus in the state of Sao Paulo	2022	ACTA TROPICA	10.1016/j.actatropica.2022.106333
Kinast, V; Klohn, M; Nocke, MK; Todt, D; Steinmann, E	Hepatitis E virus species barriers: seeking viral and host determinants	2022	CURRENT OPINION IN VIROLOGY	10.1016/j.coviro.2022.101274

Authors	Title	Publication year	Journal	DOI
Lu, Y	Textile-embedded cell-free biosensors	2022	NATURE BIOMEDICAL ENGINEERING	10.1038/s41551-022-00869-3
Slater, J	Eating Insects: A Christian Ethic of Farmed Insect Life	2022	STUDIES IN CHRISTIAN ETHICS	10.1177/09539468211045030
Fearnley L.	Agnotology of virology: The origins of Covid-19 and the next zoonotic pandemic	2022	INTERNATIONAL REVIEW OF ENVIRONMENTAL HISTORY	10.22459/IREH.08.01.2022.08
Rosa D., Villa G., Giannetta N., Dionisi S., Togni S., Bonetti L., Terzoni S., Di Muzio M., Di Simone E.	Tackling the Italian emergency 2019-nCoV: nurses' knowledge, attitudes and behaviors	2022	ACTA BIOMEDICA	10.23750/abm.v93i1.12316
Zahmanova, G; Takova, K; Valkova, R; Toneva, V; Minkov, I; Andonov, A; Lukov, GL	Plant-Derived Recombinant Vaccines against Zoonotic Viruses	2022	LIFE-BASEL	10.3390/life12020156

TABLE II

Selected opposers' arguments

Reference	Text opposing	Key issue
1	Carroll et al. emphasised that viral pandemics, such as the coronavirus disease 2019 (COVID-19), should not be defined as global syndemics, however, a syndemic perspective can conceptualise a variety of vulnerabilities caused by COVID-19.	Disagreeement with classification (syndemics)
2	Ideally, we would stop epidemics, pandemics, and other disasters from happening at all, through primary prevention targeting these multiple threats. Over the decade 2009–19, the US Agency for International Development spent US$170 million on evaluating the 'feasibility of pre-emptively mitigating pandemic threats', via the Global Virome Project (GVP). Even though a novel human pathogen is likely to be of animal origin (three-quarters are zoonotic) and most likely to be a virus, the chance of identifying the next pathogen by screening the entire global virome is small. The animal viruses that have the potential to infect people would have to be found among millions of undiscovered virus species. The search might be more fruitful if focused on especially risky pathogens, notably mammalian RNA viruses. However, not even retrospective analyses of COVID-19 have identified the pathway of coronavirus SARS-CoV-2 from animals to humans. Viral emergence from animal reservoirs is still largely unpredictable and unpreventable.	Viral emergence from animal reservoirs is still largely unpredictable and unpreventable
3	The ability to detect novel viruses has greatly improved with the decreased costs of metagenomics next-generation sequencing. It is estimated that more than half a million yet-to-be-discovered viruses with zoonotic potential are circulating in mammals and birds. This argument has been used to support the Global Virome Project, aiming to characterise these viruses before they become zoonotic. However, such undertakings have been difficult and expensive, and BASV was one of the few human viruses discovered by such projects.	Difficult and expensive
4	The discovery of new potential human pathogens is a useful strategy for EID prevention. However, given the high cost and uncertainty about its effectiveness, this strategy has been highly criticised.	High cost and inefficiency
5	In the following decade, the USAID PREDICT program, the EcoHealth Alliance, Metabiota and other research groups poured funds and resources into sampling viruses from animals, sequencing them, studying them and as- sessing their potential risk. Carroll himself spearheaded the work of a consortium called the 'Global Virome Project'— an effort to build transnational collaboration across these viral discovery programs in order to construct a map of the global virome — a 'global atlas of most of the planet's naturally occurring potentially zoonotic viruses'. But as Carroll acknowledged in his talk that day in Beijing, such an effort could not realistically sample all viruses on the planet. Rather, it would begin by focusing viral discovery on certain high-risk geographical regions, certain high-risk animal species and certain high-risk virus families	Such an effort could not realistically sample all viruses on the planet
6	Such limitations have large, uncomfortable implications for proposed preemptive surveillance programmes that rely on these measures of 'the where, when and what' of disease emergence to be transparently costed, adequately targeted and potential efficacy evaluated. Cycles of scientific advance, revision and refinement are necessary, and these are all important scientific endeavours to which we have ourselves contributed, but we can ill afford to direct scarce disease emergence-prevention resources where we risk failing to deliver appreciable gains in risk reduction or health protection	Lack of resources
7	Trust is undermined when scientists make overblown promises about disease prevention.	Overblown promises
8	New technologies, such as gene drives or predictive genomic screening, are often touted as a solution to dis- ease problems with the potential for eradication (total elimination of naturally caused human cases). However, setbacks encountered by disease eradication and elimination schemes over the past century - including a large-scale Soviet effort to purge its republics of endemic plague -starkly remind us that many such diseases require long-term multifaceted policies and programs, and even then eradication can fail.	Need long-term
9	To prevent future pandemics, the Global Virome Project was established, focusing on early identification of the pandemic-potential of viruses found in wild animals. Despite the tremendous manpower and resources required by this project, this effort alone was unable to prevent the current pandemic of SARS-CoV-2	Could not predict COVID-19
10	One criticism of the GVP is that the scale of the task is simply unmanageable. PREDICT researchers estimate that there are 1.67 million unknown viruses in mammals and birds, and although this figure is contested, there is no doubt that the virome is vast. It is also constantly changing, so one-off discovery efforts would not be enough. "RNA viruses evolve at a hefty rate," says Edward Holmes, a virologist at the University of Sydney in Australia. "So you'd have to keep doing it."	Task is simply unmanageable
11	Systematic approaches to the characterisation of viruses with potential for zoonotic spillover bear particular biosecurity risks. Large-scale efforts with the aim to collect hundreds of thousands of samples of viruses and investigate them in laboratories have been proposed and initiated. Such efforts are associated not only with accidental exposure and release risks but also the potential of generating dual-use insights. Large-scale characterisation of animal viruses may enable computational viral engineering capabilities by creating large data sets which link genetic sequence and function for thou- sands of viruses. This may be leveraged to create more transmissible and virulent pathogens. In addition, broad genomic surveys and characterisation of animal viruses have been suggested to be of little practical use to mitigate the emergence of biological events. Therefore, preferential investment into approaches which are associated with little biosecurity risk may more robustly reduce overall health security risk. For instance, the real-time surveillance of human populations for emerging pathogens does not involve large-scale collection and characterisation of zoonotic viruses and has been highlighted as an effective approach to mitigating outbreaks.	Dual-use insights, biosecurity risks, little practical use, surveillance better

Reference	Text opposing	Key issue
12	The major research investments in spillover prevention have focused on pathogen discovery in wildlife and surveillance where there is likely to be contact between wildlife hosts and people. Although understanding the diversity of wildlife pathogens in natural environments and making improvements in disease detection in high-risk human communities is essential, these approaches are insufficient to prevent spillover events.	Insufficient to prevent spillover
13	Currently, our ability to predict human infectivity among RNA viruses is limited to (mostly weak) ecological correlates, and the value of massive investment in virus discovery and sequencing programs is hotly debated.	Too costly

TABLE III

Selected supporters' arguments

Reference	Text supporting	Key issue
14	The proposed Global Virome Project would aim to spend US$1 billion over the next decade discovering a million more. Future programmes such as these present an opportunity to test model-guided approaches as both a cost-saving measure and shortcut to accelerate scientific progress	Importance of (global) surveillance efforts for prevention
15	Programs to pre-emptively identify novel pathogens that could emerge so that vaccines or therapeutics could be more broadly targeted, or to reduce the underlying drivers of emerging diseases are considered a valid part of a pre-emptive strategy	Importance of (global) surveillance efforts for prevention
16	Today, large host-parasite datasets, increased computing power, and new macroecological methods are being used to estimate symbiont biodiversity, and to set sampling targets (e.g., the Global Virome Project's proposed goal of describing 85% of mammal and bird viruses). These methods can be used to estimate how many host species need to be sampled to reach parasite description targets.	Methodological feasibility of GVP
17	Current systems are not equipped to deal with pandemics. Past epidemics with pandemic potential were mainly identified through an unusual cluster of severe cases or deaths in humans. This means of identification is weak, and is often missed by classic surveillance systems. Estimates suggest that 1.7 million viruses exist across 25 high consequence viral families, of which 500 000-700 000 are likely to be zoonotic. Few viruses are likely to have the ability to infect humans and even fewer the ability to spread. Even if the likelihood of spreading is low, the impact, as illustrated by the COVID-19 pandemic, might be disastrous and justifies investment in systems that can prevent such events.	Importance of (global) surveillance efforts for prevention
18	Metagenomics is the study of genomic material recovered directly from environmental samples. Thus, conversely to genomic samples, metagenomic samples consist of genome sequences of a community of organisms sharing the same environment, highlighting the microbial diversity in the environmental samples. The samples of genome sequences are collected using shotgun sequencing. This creates a mixture of genome fragments from all organisms in the environment. One important step in metagenomics is to assign each fragment to its owner, allowing to identify and quantify species. This step is called read assignment, and it is the basic step in most metagenomic analysis workflows such as in genomic epidemiology, and viral epidemiology.	Importance of metagenomics in viral epidemiology
19	In a recent report on pandemics and the environment and a policy paper aimed at the incoming US administration, we called for the formation of a high-level intergovernmental council on pandemic prevention and national One Health platforms to strengthen our preparedness.	Importance of (global) surveillance efforts for prevention
20	We strongly urge that scientists in these countries work to discover all of these viruses so that we can catalog them, develop a reference library for rapid pathogen identification and risk assessment, and test vaccines and therapies against them	Development of a reference database for rapid pathogen identification and risk assessment
21	However, deciphering the global virome and its structure becomes challenging not just because of the technical difficulties associated with the screening of diverse viruses, but because their many hosts are often understudied, particularly those from remote areas. The Neotropical region is one such example; being among the most biodiverse realms on Earth, Neotropical rainforests are candidates to host and conserve relevant parts of the virosphere diversity. However, the knowledge of Neotropical viral diversity and their hosts is in its infancy. Given that zoonotic viruses are considered the most probable causative agents of emerging diseases and that mammal and avian species are their main hosts, the study of the wildlife viral diversity and their ecological context in these remote areas will provide important information for public and animal health to prevent potential new emerging viral epidemics.	Importance of understanding virus diversity and ecology, as well as virus emergence processes/potential
22	Thus far, virome characterisation has been largely achieved through several independent initiatives. It is a matter of debate whether ongoing coordinated global efforts toward a Global Virome Project would be justified. Assembling, cataloging, and making publicly available the genomes of DNA and RNA viruses infecting domestic and wild hosts is an ambitious yet reachable achievement in the next two decades. The cost estimates for a 10 years global effort toward exhaustive metagenomic characterisation of the global virome are about 3-4 billion US dollars (USD). Compared with the estimated 16 trillion USD economic losses of the current pandemic, a Global Virome Project would be worth being supported even if the best expected outcome was shortening by 1 month the resolution of the next pandemic.	GVP worth being supported based on economic considerations
23	International partnerships, such as the Global Virome Project for virus hunting worldwide and the establishment of a CDC network in Africa with assistance from the US CDC and China CDC, will greatly improve our capacity for surveillance, contributing to, ideally, a worldwide system in the "big data" era of the 21st century	Importance of (global) surveillance efforts for prevention
24	Viruses that are capable of spreading by vector or airborne routes - one of the most important pandemic threats - continue to emerge. More than 1.5 million as yet unknown viruses are estimated to exist in animals worldwide, and 38 to 50% of them are candidates to spread to humans. Global-surveillance and virus-discovery programs are therefore important, and they may be able to predict pandemics.	Importance of (global) surveillance efforts for prevention

Reference	Text supporting	Key issue
25	Hence, there is an urgent need to establish a global, genomic-based biosurveillance platform, a development which would be of immense value to biosecurity, biodefense, and the economy. If implemented, this "pandemic interception system" would hugely advance our under- standing of the natural world. Three major research programs are poised to support this effort: BIOSCAN, the Earth BioGenome Project (EBP), and the Global Virome Project (GVP). Each of these global programs is now working to develop approaches in comparative genomics that are needed to discover all species and to reveal their interactions. The GVP was conceived in 2016 by researchers and policymakers from different disciplines and professional sectors around the globe in response to the repeated, unpredictable emergence and re-emergence of high-impact viral epidemics and pandemics compromising global health security and human and animal well-being. The GVP benefits from a leadership team with decades of experience in developing and implementing innovative solutions in pandemic prevention and advocating for, and working in, global emerging infectious disease research, policy, and capacity strengthening. The GVP's standard operating procedures and technological and modelling innovations have already enabled the discovery, detection, and risk characterisation of 1,200 potentially zoonotic viruses from 35 countries, including more than 100 novel coronaviruses. Its mission will be achieved through a collaborative partnership among public, private, philanthropic, and civil organisations to detect the majority of our planet's unknown virus threats to prepare for and stop future epidemics.	Development of a reference database for rapid pathogen identifications and risk assessment
26	Most emerging infectious diseases of humans are viruses that originate from nonhuman animals via "zoonotic" transmission. Given the diversity of animals and viruses in nature, targeting virus discovery, surveillance, and research toward the taxonomic groups with the highest propensity to infect humans would benefit attempts to mitigate future disease outbreaks	Importance of understanding virus diversity and ecology, as well as virus emergence processes/potential
27	There are four reasons the world needs an intergovernmental panel on pandemic risk. First, the fundamental science on pandemic risk is evolving. Knowledge in this field has advanced considerably, but there is much to learn about the range of real and potentially pathogenic hazards, the probability of future pandemics, and effective response strategies	Importance of (global) surveillance efforts for prevention
28	One health, or the concept that humans, animals, and the environmental health are intrinsically linked, has provided a lens to study possible cross-species transmission of viruses from bats to humans or other amplifying hosts. The majority of viruses is previously unknown or not represented in public sequence databases, making virome characterisation a particularly challenging task. Approximately 263 viruses from 25 families are known to infect humans, but viruses of 40,000 species are estimated to infect mammals. Of those viruses, approximately 10,000 are estimated to have zoonotic potential. Innovative advancements in unbiased high throughput sequencing (HTS), coupled with increased computational power, have broadened the capacity for viral discovery in recent years. Describing and classifying previously unknown viruses and sharing them in public sequence databases not only helps the scientific community to better understand basic biology, but can ultimately improve detection and facilitate the prediction of viral emergence, and hence helping prevention or mitigating future disease outbreaks.	Development of a reference database for rapid pathogen identifications and risk assessment; Importance of (global) surveillance efforts for prevention
29	Characterising the diversity of viruses in key wildlife species will help to reduce the time between detection and response during an outbreak. Furthermore, the global virome project was launched to detect and identify viral threats to human health, characterise the host ranges of viruses, identify behaviours that favour spillover, establish a global surveillance network and identify transmission and pathogenicity markers for high-risk viruses.	Importance of understanding virus diversity and ecology, as well as virus emergence processes/potential
30	Investigating viral diversity in wildlife reservoirs is a building block for preparedness for future epidemics. The discovery of novel viruses in animal reservoirs can improve the rapid identification of emerging pathogens and their ecological niche, allowing risk reduction strategies for spillover events and diminishing the severity of emerging outbreaks. However, as the vast majority of the wildlife virome is still unknown, hunting novel viruses remains an interminable task. Traditionally, cell culture techniques were applied for virus discovery. However, the vast number of viruses is nonculturable; thus, exploration of viral diversity necessitates culture-independent techniques, such as genomic sequencing. Carroll et al. (2018) estimated that several billion dollars would be needed to unravel all unknown viral species in mammalian and avian hosts by using genomic sequencing.	Development of a reference database for rapid pathogen identifications and risk assessment
31	These findings yield direct lessons for the growing enterprise of conducting genetic surveillance on zoonotic pathogens in their animal reservoirs. Our cross-scale analysis opens the door for a new generation of integrative risk assessment models for pathogen emergence, which will integrate growing streams of data collected in laboratories and field surveillance programs. At present, we rely on the intuition of individual scientists to link together the discoveries from targeted experiments, massively parallel phenotypic screens, experimental evolution, clinical medicine, and field epidemiology and disease ecology. Mathematical and computational models that connect biological scales using mechanistic principles can make unique contributions to this transdisciplinary enterprise, by formally integrating diverse empirical findings and by identifying the crucial knowledge gaps to focus future research. The work presented here is a step on the path to realising this potential.	Importance of (global) surveillance efforts for prevention; Methodological feasibility of GVP
32	High-throughput sequencing (HTS) already enables EID surveillance at interfaces where humans, wildlife, and domestic animals overlap, by screening for pathogens in likely reservoir hosts. Yet, we have made relatively little use of the extraordinary potential of HTS approaches to understand how climate, season, behaviour and other ecological factors affect pathogen diversity, in both the community of pathogens and the within-pathogen heterogeneity that allow for increases in virulence and infectivity. The solution is increased storage and management of pathogen genome and metagenome databases coupled with additional field and laboratory expertise to document the breadth of global pathogen diversity.	Development of a reference database for rapid pathogen identifications and risk assessment

Reference	Text supporting	Key issue
33	Secondly, we have to better understand our enemy. Brooks et al. spelled it out in an acronym, DAMA - Document, Assess, Monitor, Act. Zoonotic EIDs pose the most significant threat to our society, but the pandemic potential differs markedly between pathogens. We need to dedicate more resources to sequencing the virome of wildlife and our livestock, enabling us to better track gene flow, and assess spillover events and hybridisation. Various programmes are already in place. For example, the Global Virome Project conducts viral discovery and provide timely data for public health interventions against future pandemics. An estimated 1.67 million viral species have yet to be discovered from mammal and bird hosts, and the costs of sequencing viruses with the highest zoonotic potential may be great (1.2 to 7 billion US dollars), but they are dwarfed by the cost of another pandemic	Importance of understanding virus diversity and ecology, as well as virus emergence processes/potential
34	Recently, meta-genomic and meta-transcriptomic analyses have been increasingly used to survey the virome in diverse animals over a wide geographical area. Conclusions These findings, combined with our previous viral surveys from rodents, bats, and mosquitoes, and the online viral databases for these animals (DBatVir, DRodVir, and DMosVir, http://www.mgc.ac.cn/), greatly increase our knowledge of the viral community in wildlife and arthropod vectors in densely populated countries of East and Southeast Asia. Continued efforts in viral surveillance among wildlife hosts will reveal greater diversity of viral lineages, as recently hypothesised globally and provide evidence to mitigate the risk of potential zoonotic disease emergence, as well as build the regional and global capacity for effective prevention and response to EIDs.	Importance of (global) surveillance efforts for prevention
35	The Global Virome Project (GVP), which was proposed and initiated at the beginning of 2018, estimated that there are over 1.67 million yet-to-be-discovered viruses in animal reservoirs, while 631,000–827,000 of these un- known viruses can infect humans. Therefore, the development of rapid methods for identifying the potential human-infecting viruses is in great need. There are some limitations to this study. Firstly, the human-infecting viruses used here is far from complete when compared to those estimated by the GVP. However, the viruses used in this study covered 30 families in all groups of the Baltimore classification, which were similar to those estimated by the GVP.	Importance of understanding virus diversity and ecology, as well as virus emergence processes/potential
36	It is known that pathogens can lead to serious infectious diseases worldwide and cause enormous economic losses, thus the research of pathogens is essential to public health. Recently, antimicrobial resistance has become a serious threat to public health globally. A better understanding of the evolution and diversity of pathogens is critical for prevention and control of antimicrobial resistance.	Importance of understanding evolution and diversity of pathogens
37	Besides, our knowledge of the viral population and its ecological diversity harboured by wildlife is largely obscure. Therefore, an in-depth understanding of the spectrum of viruses existing in wildlife, in addition to their prevalence and distribution, will contribute to the prevention and control of emerging viral infectious dis- eases of wildlife origin.	Importance of understanding virus diversity and ecology, as well as virus emergence processes/potential

REFERENCES

1. Borghi-Silva A, Back GD, Garcia de Araújo AS, Oliveira MR, da Luz Goulart C, Silva RN, et al. COVID-19 seen from a syndemic perspective: Impact of unhealthy habits and future perspectives to combat these negative interactions in Latin America. Prog Cardiovasc Dis. 2022; 71: 72-8.
2. Dye C. The great health dilemma: is prevention better than cure? Oxford University Press; 2021. Available from: https://ora.ox.ac.uk/objects/uuid:11cc474b-68aa-4ad3-a608-8edd4f32ee96.
3. Edridge AWD, Abd-Elfarag G, Deijs M, Jebbink MF, van Hensbroek MB, van der Hoek L. Divergent Rhabdovirus discovered in a patient with new-onset nodding syndrome. Viruses. 2022; 14(2): 210.
4. Ellwanger JH, Kaminski VL, Chies JAB. Emerging infectious disease prevention: Where should we invest our resources and efforts? J Infect Public Health. 2019; 12(3): 313-6.
5. Fearnley L. Agnotology of virology: the origins of Covid-19 and the next zoonotic pandemic. Int Rev Environ Hist. 2022; 8(1): 121-30.
6. Fisher MC, Murray KA. Emerging infections and the integrative environment-health sciences: the road ahead. Nat Rev Microbiol. 2021; 19(3): 133-5.
7. Holmes EC, Rambaut A, Andersen KG. Pandemics: spend on surveillance, not prediction. Nature. 2018; 558(7709): 180.
8. Jones SD, Atshabar B, Schmid BV, Zuk M, Amramina A, Stenseth NChr. Living with plague: lessons from the Soviet Union's antiplague system. Proc Natl Acad Sci. 2019; 116(19): 9155-63.
9. Kawang K, Naoudom U, Kowitdamrong E, Kerr SJ, Ruxrungtham K, Nilaratanakul V. Sensitivity and specificity of anti-double-stranded RNA immunofluorescence for universal detection of viral infection in respiratory specimens. Heliyon. 2021; 7(12): e08471.
10. Makin S. Could an algorithm predict the next pandemic? Nature. 2022; 610(7933): S42-4.
11. Musunuri S, Sandbrink JB, Monrad JT, Palmer MJ, Koblentz GD. Rapid proliferation of pandemic research: implications for dual-use risks. mBio. 2021; e0186421.
12. Plowright RK, Reaser JK, Locke H, Woodley SJ, Patz JA, Becker DJ, et al. Land use-induced spillover: a call to action to safeguard environmental, animal, and human health. Lancet Planet Health. 2021; 5(4): e237-45.
13. Woolhouse M. Sources of human viruses. Science. 2018; 362(6414): 524-5.
14. Albery GF, Becker DJ, Brierley L, Brook CE, Christofferson RC, Cohen LE, et al. The science of the host-virus network. Nat Microbiol. 2021; 6(12): 1483-92.
15. Berry K, Horan RD, Finnoff D, Pompa R, Daszak P. Investing to both prevent and prepare for COVID-XX. EcoHealth. 2022; 19(1): 114-23.
16. Carlson CJ, Hopkins S, Bell KC, Doña J, Godfrey SS, Kwak ML, et al. A global parasite conservation plan. Biol Conserv. 2020; 250: 108596.
17. Carroll D, Morzaria S, Briand S, Johnson CK, Morens D, Sumption K, et al. Preventing the next pandemic: the power of a global viral surveillance network. BMJ. 2021; 372: n485.
18. Cobas D, Mäkinen V, Rossi M. Tailoring r-index for document listing towards metagenomics applications. In: Boucher C, Thankachan SV, editors. String processing and information retrieval. Cham: Springer International Publishing; 2020. p. 291-306. (Lecture Notes in Computer Science).
19. Daszak P. Lessons from COVID-19 to help prevent future pandemics. China CDC Wkly. 2021; 3(7): 132-3.
20. Daszak P, Olival KJ, Li H. A strategy to prevent future epidemics similar to the 2019-nCoV outbreak. Biosaf Health. 2020; 2(1): 6-8.
21. Fernández-Correa I, Truchado DA, Gomez-Lucia E, Doménech A, Pérez-Tris J, Schmidt-Chanasit J, et al. A novel group of avian astroviruses from neotropical passerine birds broaden the diversity and host range of Astroviridae. Sci Rep. 2019; 9(1): 9513.
22. Fulci V, Carissimi C, Laudadio I. COVID-19 and preparing for future ecological crises: hopes from metagenomics in facing current and future viral pandemic challenges. OMICS J Integr Biol. 2021; 25(6): 336-41.
23. Gao GF. From "A"IV to "Z"IKV: attacks from emerging and re-emerging pathogens. Cell. 2018; 172(6): 1157-9.
24. Gerberding JL, Haynes BF. Vaccine innovations — past and future. N Engl J Med. 2021; 384(5): 393-6.
25. Kress WJ, Mazet JAK, Hebert PDN. Opinion: intercepting pandemics through genomics. Proc Natl Acad Sci. 2020; 117(25): 13852.
26. Mollentze N, Streicker DG. Viral zoonotic risk is homogenous among taxonomic orders of mammalian and avian reservoir hosts. Proc Natl Acad Sci USA. 2020; 117(17): 9423-30.
27. Oppenheim B, Brown K, Waldman R. The world needs an intergovernmental panel on pandemic risk. Nat Med. 2021; 27(6): 934.
28. Paskey AC, Ng JHJ, Rice GK, Chia WN, Philipson CW, Foo RJH, et al. The temporal RNA virome patterns of a lesser dawn bat (*Eonycteris spelaea*) colony revealed by deep sequencing. Virus Evol. 2020; 6(1): veaa017.
29. Piret J, Boivin G. Pandemics throughout history. Front Microbiol. 2020; 11: 631736.
30. Santos PD, Ziegler U, Szillat KP, Szentiks CA, Strobel B, Skuballa J, et al. In action-an early warning system for the detection of unexpected or novel pathogens. Virus Evol. 2021; 7(2): veab085.
31. Schreiber SJ, Ke R, Loverdo C, Park M, Ahsan P, Lloyd-Smith JO. Cross-scale dynamics and the evolutionary emergence of infectious diseases. Virus Evol. 2021; 7(1): veaa105.
32. Titcomb GC, Jerde CL, Young HS. High-throughput sequencing for understanding the ecology of emerging infectious diseases at the wildlife-human interface. Front Ecol Evol. 2019; 7. Available from: https://www.frontiersin.org/articles/10.3389/fevo.2019.00126.
33. Van Oosterhout C. Mitigating the threat of emerging infectious diseases; a coevolutionary perspective. Virulence. 2021; 12(1): 1288-95.
34. Wu Z, Han Y, Liu B, Li H, Zhu G, Latinne A, et al. Decoding the RNA viromes in rodent lungs provides new insight into the origin and evolutionary patterns of rodent-borne pathogens in Mainland Southeast Asia. Microbiome. 2021; 9(1): 18.
35. Zhang Z, Cai Z, Tan Z, Lu C, Jiang T, Zhang G, et al. Rapid identification of human-infecting viruses. Transbound Emerg Dis. 2019; 66(6): 2517-22.
36. Zhou X, Zhu L, Li W, Liu Q. An integrated microfluidic chip for alginate microsphere generation and 3D cell culture. Anal Methods. 2022; 14(12): 1181-6.
37. Zhu W, Yang J, Lu S, Jin D, Pu J, Wu S, et al. RNA virus diversity in birds and small mammals from Qinghai-Tibet plateau of China. Front Microbiol. 2022; 13: 780651.
